# Prospective performance of the IWG-2023 criteria and IPSS-M in a phase 2 trial of guadecitabine for higher-risk MDS or CMML

**DOI:** 10.1016/j.bneo.2024.100008

**Published:** 2024-03-29

**Authors:** Samuel Urrutia, Prithviraj Bose, Yesid Alvarado, Gautam Borthakur, Farhad Ravandi, Naval Daver, Naveen Pemmaraju, Elias Jabbour, Koichi Takahashi, Tapan Kadia, Courtney DiNardo, Steven Kornblau, Rashmi Kanagal-Shamanna, Xuelin Huang, Kristy Bodden, Hagop Kantarjian, Guillermo Garcia-Manero

**Affiliations:** 1 Division of Cancer Medicine, The University of Texas MD Anderson Cancer Center, Houston, TX; 2 Department of Leukemia, The University of Texas MD Anderson Cancer Center, Houston, TX; 3 Department of Hematopathology, The University of Texas MD Anderson Cancer Center, Houston, TX; 4 Department of Biostatistics, The University of Texas MD Anderson Cancer Center, Houston, TX

## Abstract

Guadecitabine (SGI-110) is a dinucleotide form of decitabine that has been studied in myelodysplastic syndrome (MDS) and acute myeloid leukemia. Here, we present the results of a single-center phase 2 trial of this agent for patients with higher-risk MDS or chronic myelomonocytic leukemia (CMML). Guadecitabine was administered at a dose of 60 mg/m2 subcutaneously for 5 days. Of 100 enrolled patients, 82% had MDS. The median age was 69 years, and International Prognostic Scoring System (IPSS) was intermediate-2 in 78% and high in 14%. Thirty-eight percent had complex cytogenetics, and 32% had *TP53*^*mut*^. By the International Working Group 2006 (IWG-2006) criteria, 25% achieved complete remission (CR), 30% marrow CR, and 33% no response (NR). Common grade 3 events were febrile neutropenia (32%) and infection (25%). Mortality rates at 4 and 8 weeks were 0% and 4%, respectively. The median overall survival (mOS) was 16.8 months. Patients who underwent transplantation (21%) had an mOS of 46.6 months. We then reanalyzed this data set using IPSS-Molecular (IPSS-M) and IWG-2023 response criteria. By IPSS-M, 60% of patients were classified as very high and 27% as high risk. By IWG-2023, overall response rate was 52%, with 30% CR, 14% CR with limited count recovery, and 42% NR. IPSS-M provided adequate risk stratification at enrollment. Patients classified as marrow CR had widely different outcomes when reclassified by IWG-2023. In conclusion, SGI-110 was active in high-risk MDS, but survival is unlikely to be superior to current hypomethylating agents. The study is registered at www.ClinicalTrials.gov as #NCT02131597.

## Key Points

• Guadedecitabine was active in higher-risk MDS but unlikely to be superior to current hypomethylating agents.
• The IWG-2023 criteria responses demonstrate response-specific survival prognostication.

## Visual Abstract

**Figure:**
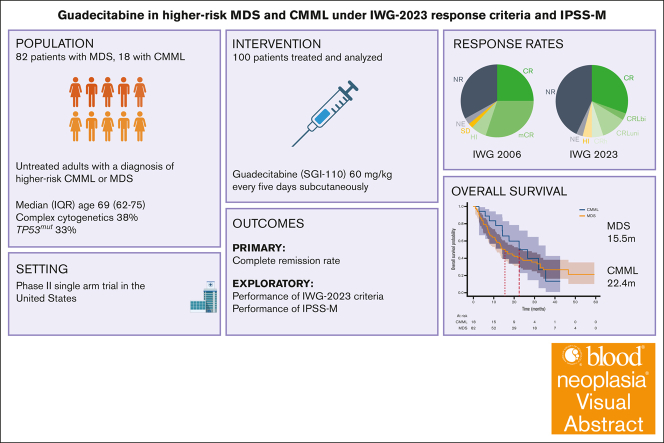

## Introduction

Myelodysplastic syndromes (MDS) are a group of bone marrow (BM) disorders characterized by hematopoietic stem cell (HSC) dysfunction, proliferation, and lack of differentiation.^1^ Their primary morphologic and clinical features are dysplasia, cytopenias, and risk of transformation to acute myeloid leukemia (AML). Patients with higher-risk MDS are treated with hypomethylating agents (HMAs).^2^ Although these agents have been shown to improve survival and improve cytopenias, long-term responses are rare.^3^ Therefore, there is a need for better agents in this disease.

HMAs are metabolized by cytidine deaminase, which may reduce their clinical effectiveness.^4^ Guadecitabine (SGI-110) is a dinucleotide form of decitabine that is subcutaneously administered with a longer half-life because of decreased degradation by cytidine deaminase.^5^ A phase 1 study established that 60 mg/m^2^ daily for 5 days was a biologically effective dose and schedule of guadecitabine in patients with AML and MDS.^5^

To increase diagnostic precision and to include prognostic molecular features, the fifth edition of the World Health Organization (WHO) classification included genomically defined entities such as MDS-SF3B1 and MDS-biTP53.^6^ Molecular observations have also led to the development of a new prognostic scoring system, known as the International Prognostic Scoring System Molecular (IPSS-M).^7^ In 2023, the International Working Group (IWG) published updated response criteria (IWG-2023) for MDS including replacing “marrow complete remissions (mCRs)” and stratifying patients who benefit from therapy at any point by creating an overall response rate (ORR) assessment.^8^ These tools have not yet been studied in prospective clinical trials. Here, we report a phase 2 trial of guadecitabine in MDS and chronic myelomonocytic leukemia (CMML) with the additional objective of evaluating the performance of the IPSS-M at enrollment and the IWG-2023 response criteria.

## Methods

### Study design and enrollment requirements

This study was a single-center, open-label, phase 2 trial at The University of Texas MD Anderson Cancer Center. Eligible patients were aged ≥18 years with a diagnosis of high-risk MDS or CMML as defined by IPSS intermediate-2 or high-risk groups or those with >10% BM blasts. Patients were required to have an Eastern Cooperative Oncology Group performance status of 0 to 2, baseline serum creatinine ≤1.5 mg/dL, aspartate transaminase or alanine transaminase ≤2.5 times the upper limit of normal, alkaline phosphatase ≤2.5 times the upper limit of normal, and total bilirubin ≤1.5 mg/dL. Patients with concurrent malignancies and pregnant persons were excluded. Birth control was mandated for patients with child-bearing potential, and patients could not undergo any chemotherapy, radiation therapy, or immunotherapy other than that specified in the protocol. Hydroxyurea was allowed for patients with elevated white blood counts. The washout period from any other therapy was 2 weeks or 30 days for investigational agents.

All patients signed informed consent, and the study was approved by the MD Anderson Cancer Center Institutional Review Board. This study was conducted according to the declaration of Helsinki and its current amendments and the International Conference on Harmonization Guidelines for Good Clinical Practice. The study protocol contains extensive details about eligibility (supplemental Material).

### Interventions and assessments

Guadecitabine was administered subcutaneously for 5 days, every 28 days, at a recommended phase 2 dose of 60 mg/m^2^.^5^ Patients were evaluated for adverse events weekly for the first cycle, then every 2 to 8 weeks. Physical examination was performed at the start of each cycle. Laboratory evaluations included complete blood counts with differential, chemistry profiles containing at least creatinine, aspartate aminotranferase (AST), alanine aminotransferase (ALT), and bilirubin. The Common Terminology Criteria for Adverse Events was used to grade adverse events. Treatment was continued until evidence of treatment failure, disease progression, unacceptable toxicity, or patient decision. Response was assessed by BM aspiration and/or biopsy at the end of cycle 1 and then every 3 cycles as clinically indicated. Cytogenetic analysis was performed at baseline and subsequently as indicated. If a patient was not in remission at this time, a definitive biopsy could be scheduled at the discretion of the treating physician but was mandated at the end of cycle 6. Response to therapy was assessed using the IWG-2006 response criteria.^9^

### Outcomes

The objective of this study was to evaluate the efficacy and safety of guadecitabine. CR was the primary outcome. Secondary outcomes included ORR, overall survival (OS), event-free survival, transformation to AML, transfusion independence, safety, and toxicity. The use of prophylactic antimicrobials was recommended according to institutional guidelines. ORR was defined as a composite of CR, mCR, or hematologic improvement (HI) at any point while on therapy after cycle 1. OS and PFS were defined as the time to death or time to death or progression after therapy initiation, respectively.

We then calculated IPSS-M scores using a publicly available tool made by the authors of this study.^7^ To evaluate the performance of the IWG-2023 criteria,^8^ we annotated new response tiers including CR with limited with bilineage recovery (CRLbi), CR with unilineage recovery (CRLuni), and CR with partial hematologic recovery (CRh). We adjudicated these response criteria to each patient using their BM and laboratory information as recorded.

### Statistical analysis

The study aimed to enroll a maximum of 100 patients. Boundaries for futility and toxicity are extensively described in the protocol (supplemental Material). Briefly, the boundary for CR rate, assuming a historical CR rate of 10% and a set target CR rate of 25% for guadecitabine, would provide the opportunity to enroll up to 90 patients before stopping the trial. Similarly, toxicity boundaries were evaluated 12 weeks after treatment initiation, and a stopping boundary of 30% with a confidence of 95% for the occurrence of grade ≥3 toxicities was set.

Summary statistics were described using Pearson χ^2^ to compare frequencies and Kruskal-Wallis test for medians. Statistical significance was set to an alpha of .05. OS was calculated using the Kaplan-Meier method and the Mantel-Cox log-rank test for intergroup survival comparison. Potential prognostic time-dependent variables or predictive variables for binary events were analyzed in univariate and multivariate Cox proportional hazards models and logistic regression models, respectively. All analyses and figures were created using R or Python software, using the statistical packages pandas and lifelines.^10^^,^^11^

## Results

### Patient characteristics

Patient baseline clinical and laboratory characteristics are presented in Table 1. From 14 November 2014 to 19 December 2018, a total of 82 patients with untreated MDS (82%) and 18 with CMML (18%) were enrolled in the trial. By IPSS, patients enrolled were classified as high (14%), intermediate-2 (78%), and intermediate-1 (8%). Thirty-eight percent of patients had complex cytogenetics, 33% had *TP53*^*mut*^, and 21% had *TP53*^*multi*-*hit*^. The most common mutations were *TP53* (37%), *ASXL1* (22%), and *DNMT3A* (18%) in MDS and *ASXL1* (61%), *TET2* (61%), *NRAS* (28%), and *SRSF2* (22*%*) in CMML (supplemental Figures 1-3).

**Table 1.** **Baseline patient characteristics**

	Overall	MDS	CMML	*P* value
n (% or IQR)	100	82	18	
Age, y	69.0 (62.5-75.0)	68.0 (60.2-74.8)	72.5 (69.0-75.8)	.057
Male	62 (62.0)	53 (64.6)	9 (50.0)	.373
Hemoglobin, g/dL	9.3 (8.4-10.1)	9.2 (8.5-10.0)	9.5 (8.3-10.9)	.555
Platelets, ×10^9^ cells per L	52.0 (21.5-104.0)	53.0 (22.0-98.0)	48.0 (21.8-194.5)	.615
ANC, ×10^9^ cells per L	0.9 (0.5-1.8)	0.8 (0.4-1.5)	2.8 (1.7-9.8)	<.001
BM blast, %	10.0 (5.0-14.8)	9.0 (4.0-14.2)	11.5 (10.0-14.2)	.039
Complex cytogenetics	38 (38.4)	36 (43.9)	2 (11.8)	.027
**Cytogenetic risk category**				
Very good		1 (1.2)		.148
Good		20 (24.7)		
Intermediate		12 (14.8)		
Poor		12 (14.8)		
Very poor		36 (44.4)		
**WHO fifth edition**				
MDS-f		2 (2.4)		<.001
MDS-IB1		14 (17.1)		
MDS-IB2		33 (40.2)		
MDS-LB		11 (13.4)		
MDS-LB-SF3B1		1 (1.2)		
MDS-biTP53		21 (25.6)		
MD-CMML			7 (38.9)	
MP-CMML			11 (61.1)	
**IPSS-R**				
Low		2 (2.5)		.283
Intermediate		8 (9.9)		
High		27 (33.3)		
Very high		44 (54.3)		
**IPSS-M**				
Low		2 (2.4)		.058
Moderate low		3 (3.7)		
Moderate high		6 (7.3)		
High		22 (26.8)		
Very high		49 (59.8)		
*TP53*∗	33 (33.7)	30 (38.5)	2 (11.8)	.068
*TP53* multihit†	21 (21.4)	21 (26.2)		
**Mutational ontology**‡				
Chromatin remodeling	8 (8.0)	6 (7.3)	2 (11.1)	<.001
DNA methylation	7 (7.0)	6 (7.3)	1 (5.6)	
Other mutations	18 (18.0)	17 (20.7)	1 (5.6)	
RAS signaling	14 (14.0)	6 (7.3)	8 (44.4)	
Spliceosome	2 (2.0)	1 (1.2)	1 (5.6)	
Transcription factors	18 (18.0)	16 (19.5)	2 (11.1)	
R. tyrosine kinase	1 (1.0)		1 (5.6)	

ANC, absolute neutrophil count; IQR, interquartile range.

∗ n = 95/100.

† Multihit status was determined as one of the following (1) *TP53* VAF ≥ 50%; (2) *TP53* + 17 or 17p deletion; or (3) ≥2 *TP53* mutations.

‡ Mutational groups as published in Ogawa 2019.^25^

### Response assessment by IWG-2006 criteria

**Figure 1.**
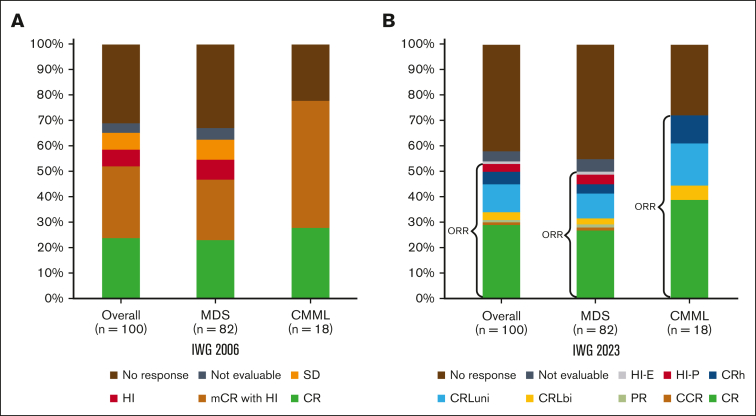
**Response assessment.** Response rates by IWG-2006 (A) and IWG-2023 (B). CRh is defined as “BM: <5% myeloblasts; and dysplasia may persist; PB: not meeting criteria for CR or CRL, no Hb threshold required, platelets ≥50 × 10^9^/L; neutrophils ≥0.5 × 10^9^/L; and blasts 0%”^8^; CRL is defined as “BM: <5% myeloblasts; and dysplasia may persist; PB: blasts 0%; CRLuni: PB, not meeting CR but only 1 of the following: Hb ≥10 g/dL; platelets ≥100 × 10^9^/L; and neutrophils ≥1.0 × 10^9^/L; CRLbi: PB, not meeting CR but only 2 of the following: Hb ≥10 g/dL; platelets ≥100 × 10^9^/L; and neutrophils ≥1.0 × 10^9^/L.”^8^ CCR, complete cytogenetic response; HI-E, HI-erythroid; HI-P, HI-platelets; PB, peripheral blood; SD, stable disease.

The median number of cycles received by patients was 5 (interquartile range, 3-8). The median number of cycles to best response was 3 (interquartile range, 2-5). The ORR was 62%: 25% CR, 30% mCR, and 7% HI alone. Two percent had stable disease, and responses were not evaluable in 3%. Thirty-three percent of patients did not respond to therapy (Figure 1; Table 3).

**Table 2.** **TEAEs**

Adverse event (>10%)	Grade 1 or 2	Grade 3	Grade 4	Grade 5
Fatigue	79	6	0	0
Nausea	37	1	0	0
Injection site reaction	29	0	0	0
Constipation	23	0	0	0
Dyspnea	22	4	0	0
Cardiac arrest	0	0	0	3
Mucositis oral	22	3	0	0
Diarrhea	14	0	0	0
Vomiting	12	1	0	0
Febrile neutropenia	0	32	0	0
Lung infection	0	25	1	2
Platelet count decreased	0	16	7	0

**Table 3.** **Response assessment by intention-to-treat, IWG-2006 and -2023 criteria**

	Overall	MDS	CMML	*P* value
**IWG-2006, n (%)**				
ORR	62 (62)	48 (59)	14 (78)	.23
CR	25 (25)	20 (24)	5 (28)	.771
mCR with HI	30 (30)	21 (26)	9 (50)	.269
HI	7 (7)	7 (9)		
Stable disease	2 (2)	2 (2)		
Not evaluable	3 (3)	3 (4)		1
No response	33 (33)	29 (35)	4 (22)	
**IWG-2023, n (% or IQR)**				
ORR	52 (52)	39 (48)	13 (72)	.102
CR	30 (30)	23 (27)	7 (39)	.608
Complete cytogenetic remission∗	1 (1)	1 (1)		.499
Partial response	1 (1)	1 (1)		
CRLbi	3 (3)	2 (2)	1 (6)	
CRLuni	11 (11)	7 (9)	3 (17)	
CRh	5 (5)	3 (4)	2 (11)	
HI-P	3 (3)	3 (4)		
HI-E	1 (1)	1 (1)		
Not evaluable	3 (3)	4 (5)		1
No response	42 (42)	37 (45)	5 (28)	
Cycles to best response	3.0 (2.0-5.0)	3.0 (2.0-5.0)	3.0 (2.0-4.0)	.871
Total cycles given	5.0 (3.0-8.0)	5.0 (3.0-7.0)	6.0 (4.0-11.0)	.077
4-wk mortality	0	0	0	
8-wk mortality	4 (4)	4 (4)		
OS, mo	16.8	15.5	22.4	.24

IQR, interquartile range.

∗ Complete cytogenetic remission is a CR equivalent but reported separately in this table.

### Toxicity

Ninety-two percent of patients experienced a grade 1 or 2 treatment emergent adverse event (TEAE). Grade 3 TEAEs were observed in 47% of patients and grade 4 in 8%. The most common TEAEs were fatigue (79% grade 1 or 2), nausea (37% grade 1), injection site reaction (29% grade 1), and constipation (23% grade 1). The most common grade 3 events were febrile neutropenia (32%), infection (25%), and thrombocytopenia (16%), as seen in Table 2. Twenty-nine percent of patients required a dose reduction to 45 mg/m^2^, 15 patients to 30 mg/m^2^, and 2 patients to 15 mg/m^2^. The most common reasons were delays in count recovery in 19%, physician decision in 9%, and infection in 1%. No patients discontinued the drug because of toxicity. The 4-week mortality rate was 0%. The 8-week mortality rate was 4%. Causes of death included cardiac arrest (2%), pneumonia with septic shock (1%), and heart failure (1%). These events were not attributed to the investigational agent. The reasons for protocol discontinuation were BM transplantation (22%), inadequate response to therapy (36%), loss of response to therapy (13%), disease progression (14%), patient or physician choice (3%), and loss of follow-up (3%).

### Survival and impact of allo-HSCT

The median follow-up time was 14.2 months. The median OS was 16.8 months for the whole cohort, 15.5 months for MDS, and 22.4 months for CMML. The median EFS was 7.2 months for the whole cohort, 6.8 months for MDS, and 8.7 months for CMML (log-rank, *P* = .14; Figure 3A). Among patients who underwent allo-HSCT, in a landmark analysis, at the median time to transplant of 5.2 months, the median OS (mOS) was 46.6 months for those who underwent transplantation and 13.6 months for those who had not (log-rank, *P* < .005; Figure 3D). For patients in mCR who underwent allo-HSCT (n = 9), the mOS duration was 51.4 months, compared with 16.9 months for those who did not (log-rank, *P* < .005). Among patients who underwent allo-HSCT and had *TP53*^*mut*^ (n = 7), the mOS duration was 22.2 months vs 7.2 months in those who did not (n = 25) undergo transplant (*P* < .005). The only surviving patient at the cutoff date in November 2023 with any *TP53*^*mut*^ had undergone transplantation.

**Figure 2.**
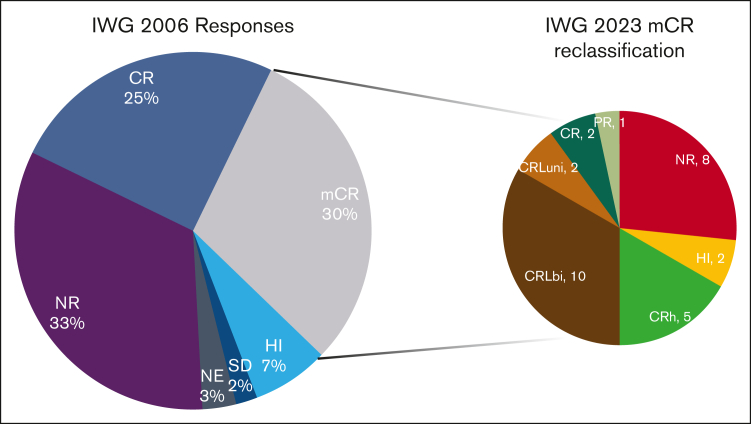
**Reclassification of marrow complete responses.** The left pie chart shows the IWG-2006 response rates. Patients with mCR were reclassified as shown in the right pie chart. Note the widely different distribution of responses from NR (8 patients) to CR (2 patients). NE, not evaluable; NR, no response.

**Figure 3.**
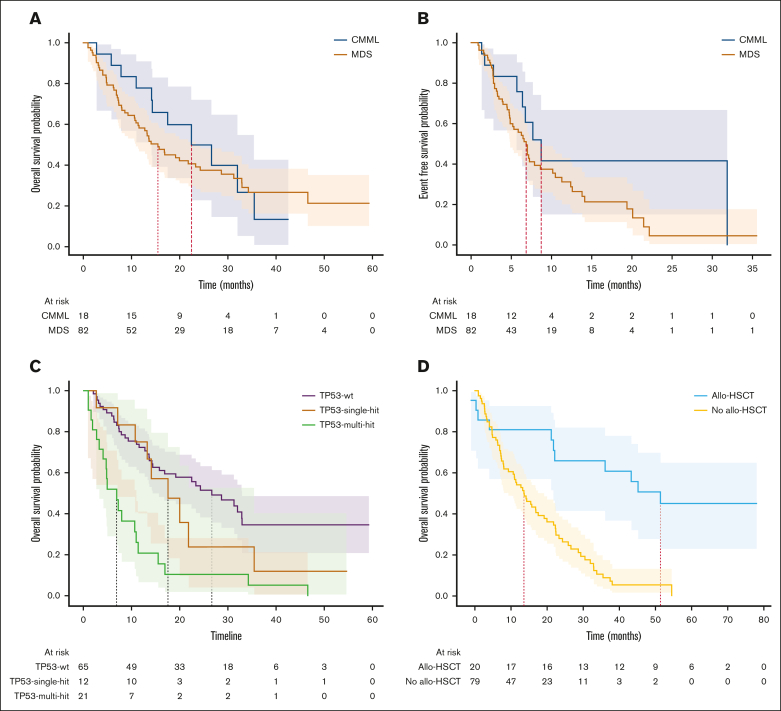
**Time-to-event analyses.** (A) OS in CMML and MDS. (B) Event-free survival by diagnosis. (C) OS by *TP53* status. (D) Landmark analysis at median time to transplant for patients who underwent allo-HSCT.

### Comparison of IPSS vs IPSS-R and IPSS-M

With conventional IPSS, patients were classified as intermediate-1 (8%), intermediate-2 (78%), or high (14%). Using IPSS-revised (IPSS-R), patients were classified as 54% very high, 33% high, 10% intermediate, and 3% low. By IPSS-M, classification was 60% very high, 27% high, 7% moderate high, 4% moderate low, and 2% low (Figure 3; supplemental Figure 6). There were no significant differences in high or very high subgroups between IPSS-R and IPSS-M (87% for both risk scores; *P* = 0.9).

### Response by IWG-2023 criteria

Under IWG-2023 criteria, the ORR was 52%, including 30% CR, 1% cytogenetic CR, and 1% partial response. Eleven percent of patients had CRLuni, 3% CRLbi, 5% CRh, and 4% HI. Three percent of patients were not evaluable, and 42% did not experience a response to therapy (Table 3). Patients in mCR by IWG-2006 (n = 30) were reclassified as CR (7%), partial response (3%), CRLbi (7%), CRLuni (33%), CRh (17%), HI (7%), and no response (27%; Figure 1).

### Impact of TP53 and the WHO fifth edition classification

There were no differences in ORR between MDS-IB1 and MDS-IB2 when the IWG-2006 criteria were applied. However, under the IWG-2023 criteria, MDS-biTP53 had a lower ORR of 33.3% than the *TP53*^*wt*^ cohort with 52% (*P* = .057). MDS-biTP53 also had a lower CR rate of 9.5% than the *TP53*^*wt*^ cohort with 32% (*P* = .012). mOS was 22.3 months in MDS-IB1 and 16.9 months in MDS-IB2 (*P* < .005). For patients with MDS-biTP53, OS was 6.8 months.

After feature selection in a univariate model, we applied a multivariate logistic regression model to predict for ORR (supplemental Figure 7; supplemental Table 1). MDSbiTP53 (odds ratio (OR), 0.15; 95% confidence interval, 0.02-0.8) and del7/7q (OR, 0.32; 95% confidence interval, 0.11-0.96) predicted for no ORR by the IWG-2023 criteria. No other WHO fifth edition entities were predictive of response or survival. In a multivariate model, MDS-biTP53 was also predictive of survival (supplemental Table 4).

## Discussion

HMAs have been the standard-of-care treatment for high-risk MDS. Azacitidine^12^ and decitabine^13^ have both resulted in blast count reduction, cytopenia correction, and increased OS duration in randomized phase 3 studies but do not result in long-term survival, and patients invariably relapse.^14^

The evaluation of response to HMA clinical activity has been standardized by the application of clinical criteria. The Cancer and Leukemia Group B (CALGB) trials^4^^,^^12^ developed response criteria in 1997 that were focused on blast clearance on visual examination of the BM and peripheral blood and normalization of hemoglobin (>13.3 g/dL for males and >11.7 g/dL for females), ANC >1.8 × 10^9^/L, and platelets >139 × 10^9^/L. Recognizing the need for standardization, response criteria were established by the IWG in 2000^15^ and later revised in 2006,^9^ 2018,^16^ and 2023.^8^ These criteria have evolved through time to include characteristics that were found in clinical trials to be of prognostic importance, such as blast count reduction, disappearance of peripheral blasts, cytogenetic abnormality clearance, and different cutoffs for improvements in cytopenia. These cutoffs have been revised extensively in new editions.

In high-risk MDS, the 2023 response criteria introduced several changes compared with prior revisions: (1) redefinition of CR with a BM blast count ≤5%, a hemoglobin cutoff of ≥10 g/dL, platelets ≥100 × 10^9^, and ANC ≥1 × 10^9^; (2) elimination of “mCR” and reclassification as limited or incomplete CR (CRL and CRh) by the number and depth of lineages improved with blast count reduction; (3) elimination of stable disease; and (4) development of an ORR by including CR, CRL, CRh, PR, and HI. These changes were based on clinical trial observations of favorable long-term outcomes in patients with any of the categories included in ORR^17^^,^^18^ and poor outcomes in patients who had blast clearance without blood count improvement,^19^ who are now considered nonresponders.

Responses by historical criteria are presented in supplemental Table 5 for azacitidine, decitabine, and guadecitabine (SGI-110), as reported in this study. Since the reduction in neutrophil and platelet counts between 2000 and 2006, the CR rates increased for both drugs. With the reduction in hemoglobin threshold, guadecitabine resulted in 4 more CRs than that of the 2006 criteria. ORRs remained mostly unchanged after being eliminated in the 2006 edition. The main difference lies in the 2006 category known as mCR, which has now been distributed between CRL and CRh. Both responses in the IWG-2006 and IWG-2023 strata retained prognostic significance. A fundamental finding of this study is the reclassification of patients who achieved mCR by IWG-2006. These patients were reclassified widely along the IWG-2023 spectrum (Figure 2) with many being reclassified as no response or complete response. Given the significantly different survival between these patients (supplemental Figure 8), the OS performance of the new criteria in future clinical trials will better stratify patients who benefit from therapy and have longer survival.

Eligibility criteria for enrollment were based on IPSS. We reclassified patients by IPSS-R and IPSS-M to demonstrate the evolution of prognostication systems. Despite most patients being of intermediate-2 risk by IPSS, we observed that by IPSS-R and IPSS-M more than two-thirds of patients were of high or very high risk (supplemental Figure 5). This sheds light into the future use of IPSS-M as a tool for clinical trial enrollment.

### Predictors of response and survival

We report that multihit *TP53* mutations (and by consequence, MDS-biTP53, as defined by the WHO fifth edition) were predictive of ORR by multivariate logistic regression analysis (supplemental Figure 7; supplemental Table 1). Patients with MDS and *TP53* mutations had similar response and HMA CR rates to those of patients without these mutations, which contrasts with a previous finding showing similar responses.^20^ As shown in supplemental Figure 7, when all patients with *TP53* mutations were pooled, there were no differences in response, but in the *TP53*^*multi-hit*^ subgroup, there was a statistically significantly higher likelihood of not achieving a response. If HMAs demethylate promoters that induce apoptosis by enhancing the activity of tumor-suppressor genes such as *TP53*, they may require the existence of at least 1 functional allele to perform their mechanism of action, a characteristic that is lacking in MDS-biTP53; without this function, the effect on hematopoiesis is minimal and survival, dismal. This finding is unique to ORR from the IWG-2023 criteria and was not observed when the same analysis was run for IWG-2006 (supplemental Table 2), suggesting that patients with mCR who had no or minimal HI had a marginal benefit from therapy and no improved survival, despite blast clearance.

Twenty-one patients in this study successfully received allogeneic stem cell transplantation. A landmark analysis shows the unmistakable benefit of transplant in this high-risk population. However, for those with *TP53*^mut^, allelic burden notwithstanding, only 1 survivor is reported here. Even with transplantation, there is a need for better therapies with deeper disease-modifying effects.

This study is limited by the post hoc adjudication of response rates using new criteria. It was also a single-center study, and although this compound is very similar to approved HMAs, slight differences in pharmacokinetics and tolerance may exist. However, it was an extensively annotated, prospective clinical trial, and to our knowledge, it was the first to evaluate the WHO fifth edition, IPSS-M, and IWG-2023 response criteria in a prospective setting.

### Effect on clinical trial design

With many late-stage clinical trials in MDS falling short of their predefined end points,^21^, ^22^, ^23^, ^24^ we cannot help but wonder whether the inadvertent inclusion of patients with *TP53*^*multi-hit*^ mutations may have skewed response rates, leading to negative results. As we continue to develop trials for this disease, enrollment stratification should account for MDS-biTP53 in efficacy analyses. Ideally, these patients should be enrolled in specific biology-informed trials. As shown in this trial with a CR of 32%, it is possible that investigational agents that have now been discounted had clinical benefit in patients with *TP53*^*wt*^, affecting access to effective drugs for patients and decreasing our armamentarium for this disease.

In this final report of guadecitabine in MDS, we demonstrated that IPSS-M is a useful tool for stratifying higher-risk populations, the molecularly defined MDS-biTP53 may be predictive of response to HMA therapy, and the IWG-2023 criteria improve the disambiguation of “less than CR” responses, with robust survival prognostication. We also demonstrated that although HSCT has a clear role in patients with MDS with no *TP53* mutations, survival is greatly diminished in those with *TP53*^*multi*-*hit*^ mutations. Designing specific trials for MDS bi-TP53 or stratifying by *TP53* allelic state will result in tailored strategies, precise effect sizes, and new therapies.

Conflict-of-interest disclosure: G.G.-M. has received research funding and honoraria from 10.13039/100013870Astex/10.13039/100019120Otsuka. The remaining authors declare no competing financial interests.

## Supplementary Material

Supplemental Data (Study Protocol)
