# Supplementary material for: Prospective performance of the IWG-2023 criteria and IPSS-M in a phase 2 trial of guadecitabine for higher-risk MDS or CMML
Source: Blood Neoplasia. 2024 Mar 29;1(2):100008. doi: 10.1016/j.bneo.2024.100008 (PMC12082099; doi:10.1016/j.bneo.2024.100008)
Supplement: Supplemental Data (Study Protocol) [file BNEO_NEO-2024-000211-mmc1.pdf]

Phase 2 Study of SGI-110 in Patients with Higher Risk MDS

2013-0901

**Short Title**	SGI-110 in Higher Risk MDS
**Study Chair:**	Guillermo Garcia-Manero
**Additional Contact:**	Xuelin Huang, Graciela Nogueras-Gonzalez
**Department:**	Leukemia
**Phone:**	713-745-3428
**Unit:**	428
**Study Co-Chair(s):**	Guillermo Garcia-Manero
**Protocol Type:**	Standard Protocol
**Protocol Phase:**	Phase II

Contents

1.0 Objectives 3
2.0 Background 3
3.0 Patient Eligibility 11
4.0 Treatment Plan 12
5.0 Pretreatment Evaluations 14
6.0 Evaluation during Study 14
7.0 Criteria for Response 15
8.0 Criteria for Removal from the Study 15
9.0 Statistical Considerations 16
10.0 Reporting Requirements 18
11. References 21

1.0 Objectives

Primary objective:

1. To evaluate the complete response (CR) rate with SGI-110 in patients with higher risk myelodysplastic syndrome (MDS).

Secondary objectives:

1. Overall response rate, survival, transformation to AML, transfusion independence
2. Safety and toxicity

2.0 Background

2.1 Myelodysplastic syndromes (MDS)

MDS is a heterogenous group of hematopoietic stem cell disorders characterized by dysplastic changes in myeloid, erythroid, and megakaryocytic progenitors, and associated with cytopenias affecting one or more of the three lineages (1-4). Patients often present with complications related to anemia (fatigue), neutropenia (infections), or thrombocytopenia (bleeding). In addition, variable blast expansion and, less commonly, leukocytosis are observed. MDS may evolve into acute myeloid leukemia (AML) in 10% to70% of patients.

The French-American-British (FAB) classification system recognizes four distinct subtypes of MDS, in addition to chronic myelomonoyctic leukemia (CMML), based on morphology and percentage of marrow blasts: RA, RARS, RAEB, RAEB-t. Prognosis of MDS patients is poor. Patients die either from complications associated with cytopenias (infections and bleeding) or transformation to AML. In practice, "low-risk" MDS may be distinguished from "high-risk" MDS by the degree of pre-leukemic blast expansion together with the combination of cytogenetic changes and degree of cytopenia. These factors have allowed the establishment of an International Prognostic Scoring System (IPSS) to predict survival and progression to AML (5).

Table 1. IPSS for MDS - Model

			**Score**		
**Variable**	0	0.5	1.0	1.5	2.0
BM blasts (%)	< 5	5-10	-	11-20	21-30
Karyotype *	Good	Intermediate	Poor		
Cytopenias	0/1	2/3			

Score for risk groups: Low, 0; INT-1, 0.5-1.0; INT-2, 1.5-2.0; High > 2.0

* Good, normal, -Y, del(5q), del(20q); poor; complex >/= 3 abnormalities, or chromosome 7 abnormalities; intermediate, other abnormalities.

Based on this scoring system, patients with low-or intermediate-1 risk MDS (approximately 70% of patients) have an expected median survival of 3.5 to 5.7 years. Median survival for patients with intermediate-2 and high-risk MDS ranges from 0.4 - 1.2 years.

2.2 The Treatment – Hypomethylating agents or "epigenetic therapy"

The standard for MDS therapy for many years has been supportive care (2,4). With the exception for allogeneic transplant, no modality has been shown to affect the outcome of patients with MDS. Intensive chemotherapy was associated with CR rates of 40% to 60%, induction mortality rates of 10% to 40% and no improvement in survival (4). The introduction of hypomethylating agents has changed the outlook of therapy for MDS. Azacitidine was given to 99 patients with MDS and compared with observation (n=92); 67% had advanced-stage disease by FAB. The response rate on the treatment arm was 60%: 7% CR and 16% PR, 37% HI. This was compared to 7% HI with observation (p<.001). The median time to leukemic transformation or death was 21 months on treatment and 13 months on observation (p=.007). Median survival was 20 months for azacitidine and 14 months for the control (p=.1). Quality of life measures were higher for azacitidine. Azacitidine was relatively well tolerated, although side effects included cytopenias and one treatment-related death. The results of this study led to approval of azacitidine in MDS and confirmed a role for hypomethylating agents in this disease (6). Similar data were noted with a second randomized study of azacitidine versus conventional care in Europe (7).

Decitabine is a deoxycytidine analog, which is phosphorylated to its nucleotide and incorporated into DNA. Once incorporated, it covalently binds to DNA-methyltransferases and traps the enzyme thus acting as an irreversible inhibitor of DNA-methyltransferase. Decitabine produces marked DNA hypomethylation (superior to azacitidine in this effect) via inhibition of DNA methyltransferase (8). At high doses, decitabine appears to cause DNA synthesis arrest due to the formation of a DNA/DNA- methyltransferase adduct, which results in cytotoxicity and apoptosis. At low doses, minimal cytotoxicty is observed, and treated cells exhibit marked reduction in DNA-methyltransferase activity, reduced overall and gene-specific DNA methylation and reactivation of silenced genes. This is associated with tumor suppressor gene activation, induction of cellular differentiation, and inhibition of clonogenic growth of leukemic progenitors.

Based on in-vitro data suggesting greater hypomethylating activity at lower doses, a phase 1 biological study of decitabine was initiated at MD Anderson. To maximize the hypomethylating effects of decitabine, multiple low dose schedules in patients with relapsed/refractory myeloid malignancies were tried. Initially, patients were treated at 5 mg/m2 iv over 1 hour daily for 10 days (dose 30-fold lower than the reported MTD). The dose was then escalated to 10, 15, and 20 mg/m2 daily for 10 days. Finally, a group of patients received 15 mg/m2 daily for 15 days then 20 days. A total of 48 patients were enrolled on the study. Responses were seen at all dose levels, but 15 mg/m2 appeared to induce the most responses, with no further benefit for increasing the dose or duration of administration. There were 9 complete remissions (CR 18%) and 7 partial remissions (14%), for a response rate of 32%. Responses were seen in refractory/relapsed AML (8/37 = 22%), MDS (4/7 = 57%), and CML (4/5 = 80%). In some patients who responded, there was a gradual diminution of blasts over 2-4 weeks, and eventual recovery of normal hematopoiesis at 5-6 weeks, suggesting a non-cytotoxic mode of action for this regimen. Response duration ranged from 2 months to 10+ months. Therefore low-dose decitabine was established as effective in myeloid malignancies, and appeared to induce remissions in part through epigenetic modulation rather than cytotoxicity.

Wijermans et al. updated the results of 169 patients with MDS treated on multiple studies with decitabine 45-50 mg/m2 iv (continuous infusion, or over 4 hours q 8 hours) daily x 3 (135-150 mg/m2 per course) every 4-8 weeks (8). The CR rate was 20%, the overall response rate was 50%, the induction mortality was 8%. The median response duration was 9 months; the median survival was 15 months. There was no difference in response rate by risk group (Table 2)

Table 2. Decitabine - response rate in MDS by risk group

IPSS Risk Group	% Response	Median Response Duration (mos)	Median Survival (mos)/% 2-yr.
Intermediate-1	46	13	23/49
Intermediate-2	52	9	15/25
High	51	8	12/26

A study was conducted in the USA and Canada, randomizing patients to decitabine + supportive care vs. supportive care alone in MDS (intermediate-1 and above). This study showed a response rate to decitabine of 17% (9% CR, 8% PR) vs. 0% in the supportive care arm, and improvements in time to AML progression and quality of life (10). This led to approval of decitabine by the FDA. A similar randomized study in Europe of decitabine versus standard of care showed decitabine to be associated with higher response rates and longer EFS; however survival was not improved (11). This was attributed to the suboptimal schedule of decitabine used, the low number of courses given, and the treatment design limiting the total number of decitabine courses to a maximum of 8.

We have completed a randomized study of 3 dose schedules of decitabine 100 mg/m2/course in MDS. Adults with advanced MDS or chronic myelomonocytic leukemia (CMML) were randomized to one of 3 decitabine schedules: 1) 20 mg/m2 IV daily x 5; 2) 20 mg/m2 sc daily x 5; and 3) 10 mg/m2 iv daily x 10. Randomization followed a Bayesian adaptive design. Ninety-five patients were treated (77 with MDS, all with IPSS score > 1.0, 18 with CMML). Overall 32 patients (34%) achieved CR, and 69 (72%) had an objective response by the new modified International Working Group criteria. The 5-day iv schedule, which had the highest dose-intensity, was selected as optimal; the CR rate in that arm was 39%, compared to 21% in the 5-day sc arm and 24% in the 10 day iv arm (p<.05) (12). The efficacy of the new schedule was confirmed in a second study in the US (13).

2.3 SGI-110

Further information is available in the Investigator Brochure.

2.3.1 General Information

The active metabolite of SGI-110 (2’-deoxy-5-azacytidylyl-(3’→5’)-2’-deoxyguanosine sodium salt), a dinucleotide, is decitabine. SGI-110 is resistant to modification by cytidine deaminase, a common pathway of decitabine metabolism and deactivation. The molecular weight of SGI-110 and decitabine are 580 Da and 228 Da, respectively. Therefore, the molar equivalent dose of 1 mg of decitabine is approximately 2.5 mg of SGI-110. SGI- 110’s activity was demonstrated with the same preclinical pharmacodynamic assays used to demonstrate decitabine’s efficacy eg, re-expression of p15, p16, and MLH1 and induction of fetal hemoglobin, in vivo. In xenograft studies, SGI-110 demonstrates promising preclinical activity in both hematologic and solid tumors.

In vitro evidence suggests that SGI-110 has a longer half-life than decitabine in the presence of cytidine deaminase. Exploratory preclinical studies demonstrate that unlike decitabine, SGI-110 can suppress the polycomb repressor complex 2 which is involved in silencing tumor suppressor genes. These promising observations suggest that SGI-110 has improved pharmaceutical properties and biological activities that expand on decitabine’s current clinical utility. SGI-110 has shown to be better tolerated in mice than decitabine and is as effective in vivo in inducing p16 expression, reducing DNA methylation at the p16 promotor region, and retarding EJ6 human bladder cancer tumor growth in athymic mice.

2.3.2 Summary of Nonclinical Data

a. In Vitro Pharmacology

The ability of SGI-110 to change global methylation status was tested by determining the methylation level of long interspersed nucleotide element-1 (LINE-1) and p16 sequences (Figure 1). Repetitive DNA elements, such as LINE-1 retro-transposable elements, serve as useful markers of genome-wide methylation changes and have previously been shown to be demethylated upon treatment with SGI-110 or decitabine (5-Aza-CdR). Both azacitidine and decitabine by itself have low objective response rates.

Figure 1: Effects of SGI-110 (filled bars) and decitabine (open bars) on LINE-1 and p16 Gene Methylation Levels in T-24 and HCT116 Cell Lines

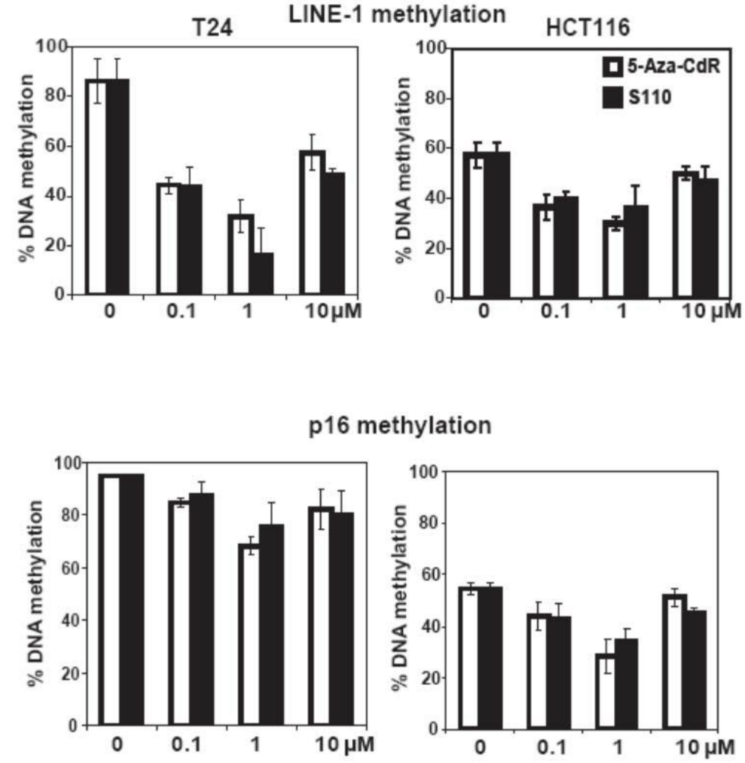

In both T-24 and HCT116 cells, the decrease in the level of methylation was dose-dependent and comparable for SGI-110 and decitabine after 0.1 µM and 1 µM treatment (Figure 1). In the figure noted above and any subsequent places in this document, S110 is the same as SGI-110. At 10 µM concentrations, only a small decrease in methylation was noted, probably due to side effects of high drug concentrations. In fact, 10 µM treatments may be too cytotoxic for effective demethylation to take place as the plating efficiency of T-24 cells indicates. It is well-established that the cytotoxic dose of these demethylating agents is not ideal for optimal epigenetic therapy, since these drugs inhibit DNA methylation best at low doses in cell lines as well as in the clinic.

Next, the changes in a methylation-silenced tumor suppressor gene, p16 were assayed in both cancer cell lines.

Figure 2: Effects of SGI-110 (filled bars) and decitabine (open bars) on p16 Gene: Expression Levels in T-24 (left) and HCT116 (right) Cells

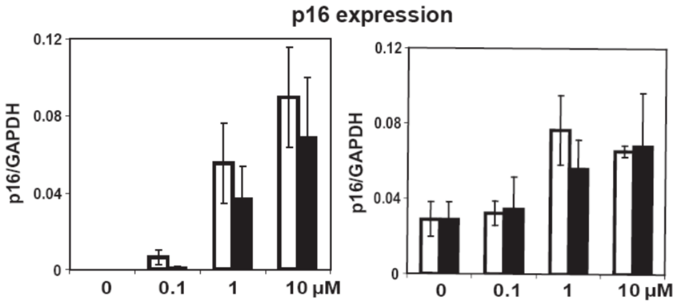

As shown in Figure 2, untreated T-24 bladder carcinoma cells do not express p16, and dose dependent increases in p16 expression were observed after 6 days of continuous treatment with SGI-110 or decitabine. After HCT116 colorectal carcinoma cells were treated for six days, a dose dependent increase in p16 expression was observed with both SGI-110 and decitabine.

In Vivo Pharmacology

The efficacy and safety of SGI-110 was evaluated in several solid tumor models and HL-60 promyelocytic leukemia in comparison to equivalent doses of decitabine (Astex Pharmaceuticals, Inc., data on file). Female nu/nu mice were implanted subcutaneously with HL-60 cells. Animals with exponentially growing tumors were randomized into 5 groups of 8 animals each to include vehicle, SGI-110 5 mg/kg administered subcutaneously every 7 days and 2.5 mg/kg administered intraperitoneally Monday to Friday. Equivalent doses and schedules of decitabine 12.2 mg/kg and 6.1 mg/kg, respectively, were administered subcutaneously for comparison. Results from this study are shown in Table 1 and Figure 3. Both SGI-110 and decitabine demonstrated equivalent antiproliferative activity when administered subcutaneously. When dosed intraperitoneally, SGI-110 seemed to have a greater effect but at the expense of higher toxicity as evidenced by more weight loss and death of 1 animal in the decitabine group.

Figure 3: SGI-110 and SGI-110 Activity and Body Weight Loss in HL-60 Promyelocytic Leukemia

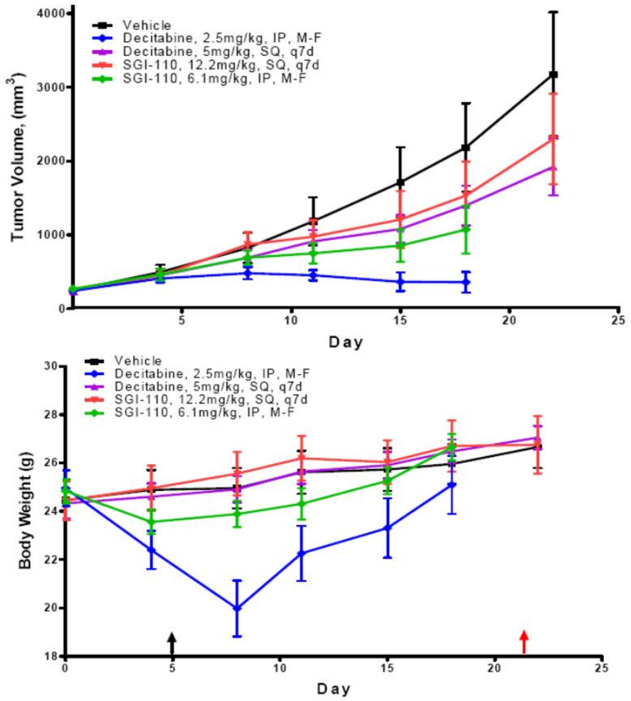

C. General Safety (Cardiac, CNS, and Respiratory)

SGI-110 was tested to examine the in vitro effects on the human ether-a-go-go related gene (hERG) potassium channel current. Whole cell patch clamp recordings were made on human embryonic kidney (HEK293) cells that were stably transfected with hERG cDNA. SGI-110 was tested at 10 µM and 300 µM.

SGI-110 inhibited hERG current by 1.4 ± 0.3% (mean ± SEM) at 10 µM and by 1.0 ± 1.3% 300 µM. The half maximal inhibitory concentration (IC50) for the inhibitory effect of SGI-110 on hERG current was not calculated but was estimated to be greater than 300 µM.

The potential neurobehavioral toxicity of SGI-110 was studied after a single subcutaneous dose of SGI-110 (0, 5, 10, and 20 mg/kg) in 2 repeat dose GLP toxicology studies in rats. Functional Observational Battery (FOB) evaluations were conducted on 10 main study animals/group pre-dose (Day -1) and 1 hour post-dose on Day 1 of the study [23] [24]. There were no SGI-110 related changes in any of the FOB measurements in either study. The noobserved-adverse-effect level (NOAEL) of SGI-110 on neurobehavioral function is 30 mg/kg.

The potential effects of SGI-110 on respiratory function were studied in a GLP study in rats. Pulmonary function (respiratory rate, tidal volume, and minute volume) were monitored continuously on 8 animals/sex/group for at least 1 hour pre-dose and at least 4 hours post-dose. Test article or vehicle was administered to all groups via a single subcutaneous injection (0, 15, 30, and 60 mg/kg) SGI-110 did not produce mortality and had no effect on clinical signs, respiratory rate, tidal volume, and minute volume. With respect to the basic pulmonary endpoints evaluated in this study, a no-observed-effect-level (NOEL) of at least 60 mg/kg has been established for SGI-110.

2.3.3 Summary of Clinical Data

For the most updated clinical data, please refer to the most recent Investigators' Brochure.

In a Phase I study, 78 patients (64 AML, 14 MDS) were enrolled in the dose escalation part: 44 patients in the daily x 5 regimen and 34 in the weekly x 3 regimen. There median age was 69 years. Median number of prior regimens was 3.

The PK profile demonstrated efficient conversion of SGI-110 to decitabine as predicted from the SGI-110 rational design, resulting in longer decitabine exposure window (beyond 8 hours) compared to Dacogen IV (3-4 hours). At SGI-110 dose range of 60-125 mg/m2, observed mean decitabine AUCs (88-231 ng*hr/mL) reach or exceed the therapeutic range seen with 20 mg/m2 Dacogen IV (115 ng*hr/mL) while achieving only a small fraction of the Cmax (26-64 ng/mL vs 146 ng/mL for Dacogen IV). The effective half-life for decitabine after subcutaneous SGI-110 injection appeared to be prolonged (up to 4-fold or ~2.4 hours) compared to Dacogen IV (0.58 hrs). SGI-110 exposures (AUC) increased in a dose proportional manner regardless of the regimen and no accumulation was observed.

Dose-related LINE-1 hypomethylation was observed in patients treated with the daily regimen between 18 and 60 mg/m2; a plateau in maximum average hypomethylation (~25%) was evident at higher daily doses (90-125 mg/m2) and therefore the BED for the daily x 5 schedule is established at 60 mg/m2. The 25% average hypomethylation of LINE-1 compares favorably with that observed historically after Dacogen IV at the dose of 20 mg/m2 daily x 5. The extent of LINE-1 hypomethylation after weekly x 3 SGI-110 was inferior as the maximum average hypomethylation plateaued at ~8% from baseline.

Starting at 36 mg/m2 daily and 60 mg/m2 weekly (44 AML, and 7 MDS patients), clinical responses were observed: 2 CRs, 1 CRp, and 1 CRi in heavily pretreated AML patients; 1 mCR and 1 HI in MDS patients previously treated with azacitidine. All responses were in patients who achieved >10% LINE-1 hypomethylation. The most common adverse events (AEs), regardless of relationship to SGI-110, were diarrhea (21%), febrile neutropenia (17%), fatigue/injection site pain/nausea at 15% each. The most common drug-related AEs were injection site pain (15%), fatigue (8%), nausea (6%), and thrombocytopenia (5%). The MTD was not reached with the weekly regimen up to 125 mg/m2 weekly x 3. With the daily regimen, 125 mg/m2 daily x 5 resulted in 2 dose-limiting toxicities (DLTs) of febrile neutropenia in 3 MDS patients (1 associated with bacteremia, and the other with sepsis and thrombocytopenia Grade 4) while none of the 9 patients with AML had DLT at that dose (14). Since the abstract publication, maximum hypomethylation was noted at the 60mg/m2 dose, while clinical MTD was defined at 90mg/m2 dose. Therefore the Phase II study proceeded to compare 60 versus 90mg/m2 daily for 5 days in newly diagnosed MDS and AML. A further arm investigated SGI-110 60mg/m2 daily x 10 days in AML salvage. Finally SGI-110 60mg/m2 daily x 5 is being evaluated in patients with MDS and progression on azacitidine/decitabine therapy.

The Phase II randomized study of the biologic effective dose 60mg/m2 daily x 5 and clinical Phase II dose 90mg/m2 daily x 5 is ongoing. As of June 30, 2013, sixty-seven patients (50 relapsed/refractory AML, 17 treatment naïve elderly AML) were treated and had a minimum follow up of 3 months. Patients were randomized to either 60 mg/m^2 dose (32 patients) or 90 mg/m^2 dose (35 patients). The median age was 66 years (range, 22–84), 69% were male, and ECOG PS of 0/1/2 was reported in 11/47/9 patients respectively. Median number of prior regimens was 2 (range, 0–10). Patients' characteristics were well balanced between the 2 dose groups. The primary endpoint of overall remissions (CR, CRp, or CRi) was observed in 17/67 patients (25% with 95% CI, 16–37%). There were 8 complete remissions (CR, CRp, or CRi) in 50 patients with relapsed/refractory AML (16% with 95% CI, 7-29%); and 9 complete remissions (CR, CRp, or CRi) in 17 treatment-naïve elderly AML patients (53% with 95% CI, 28-77%). Five patients (4 relapsed/refractory, and one treatment-naïve elderly AML) subsequently received a stem cell transplant. There was no difference in the complete remission rate between 60 and 90 mg/m^2 doses (8 remissions in 32 patients at 60 mg/m^2, and 9 remissions in 35 patients at 90 mg/m^2). LINE-1 DNA methylation data before and after treatment was available in 50 (75%) patients enrolled. LINE-1 demethylation ≥ 10% post treatment was observed in 83% and 78% in the 60 mg/m^2 and 90 mg/m^2, respectively. The median maximum LINE-1 demethylation for responders is 25% as compared to 19% for non-responders. The most common adverse events (AEs) regardless of relationship to SGI-110 ≥ Grade 3 include febrile neutropenia, thrombocytopenia, anemia, leukopenia, neutropenia, and pneumonia. The

90 mg/m^2 dose showed a greater frequency of Grade 3/4 related AEs ≥ 10% (anemia, febrile neutropenia, leukopenia, neutropenia, and thrombocytopenia) compared to the 60 mg/m^2 dose (15).

2.3.4 Risks of SGI-110 Based on Early Clinical Data

For the most up to date clinical safety information, please refer to the most recent Investigators' Brochure.

The most common risks of SGI-110 are similar to decitabine. These include myelosuppression (neutropenia, febrile neutropenia, thrombocytopenia, and anemia) and its consequences such as fever, infection, sepsis, bacteremia, or bleeding. While in GLP toxicity studies with SGI-110 subcutaneous injections, no adverse local site reactions were noted in the multiple-dose rat and rabbit studies, clinical data indicate injection site pain, irritation, or inflammation in approximately 15% of patients. Local pain seems to be ameliorated by the use of ice packs before or after injection, injecting SGI-110 slowly instead of a push, and carefully avoiding intradermal injections.

2.3.5 Potential Benefits of SGI-110

Astex Pharmaceuticals has synthesized more stable and potent inhibitors of DNA methylation than decitabine, and demonstrated that short oligonucleotides containing an azapyrimidine effectively inhibit DNA methylation in living cells. SGI-110 was synthesized by coupling decitabine and guanosine into a dinucleotide in an attempt to improve the biological stability and thereby increase the in vivo efficacy of decitabine. Unlike decitabine, SGI-110 initially is resistant to deamination by cytidine deaminases until it is converted into decitabine as a result of cleavage of the phosphodiester linkage by phosphodiesterases. As such, decitabine is the active metabolite of SGI-110. SGI-110 is a new chemical entity that may possess enhanced pharmacokinetic or pharmacodynamic properties compared to decitabine.

The activity of SGI-110 was demonstrated with the same preclinical pharmacodynamics assays used to demonstrate the activity of decitabine, e.g., re-expression of p15, p16, and MLH1 and induction of fetal hemoglobin, in vivo. In vivo data demonstrate interspecies differences with respect to absorption, distribution, and conversion to decitabine. In xenograft studies, SGI-110 demonstrates promising nonclinical activity in hematologic malignancy and solid tumors.

As such, SGI-110 is an agent that holds promising activity in hematological malignancies given decitabine's proven activity in MDS and AML. The dosage form of SGI-110 developed for use in this study as a subcutaneous injection has the potential for a more sustained release effect compared to an IV short infusion which, in addition of being more convenient, may prolong efficacy, lower toxicity and change the PK in a beneficial way.

2.4 Rationale for Proposed Schedule

The best approach for patients with higher-risk MDS remains to be defined. Both azacitidine and decitabine by themselves have low objective response rates, and a modest and limited survival benefit. From the early phase 1-2 experiences with SGI-110, it appears to be potentially more effective and perhaps safer than azacitidine and decitabine. We would like to expand our institutional experience with SGI-110 in newly diagnosed higher risk MDS to define more precisely the rates of CR, overall response, duration of response, survival and toxicity profiles. We will also assess determinants of better outcome with SGI-110 in MDS. The schedule chosen is based on the Phase II expansion of SGI-110 in higher risk MDS which treated a limited number of 40 patients. We will use SGI-110 60mg/m2 subcutaneously daily x 5 every 4 weeks. We would like to gain experience on the efficacy, outcome and safety in 100 patients with newly diagnosed MDS. The primary endpoint of the study is the objective CR rate. Secondary endpoints are overall response rate, rates of survival and transformation to AML, and toxicity profile.

3.0 Patient Eligibility

Inclusion:

1) Patients with higher risk MDS (IPSS int-2 or high; or >/= 10% blasts as defined by WHO).
- No prior intensive chemotherapy or high-dose cytarabine (>/= 1 g/m2).
- Prior biologic therapies (</= 1 cycle of prior decitabine or azacitidine), targeted therapies, or single agent chemotherapy is allowed.
- Off chemotherapy for 2 weeks prior to entering this study with no toxic effects of that therapy, unless there is evidence of rapidly progressive disease.
- Hydroxyurea is permitted for control of counts prior to treatment.
- Hematopoietic growth factors are allowed.

2) Age >/= 18 years.

3) ECOG performance status </= 2.

4) Have adequate renal function (serum creatinine </= 1.5 mg/dL)

5) Serum bilirubin </= 1.5 x upper limit of normal (ULN)

6) Aspartate transaminase (AST) or alanine transaminase (ALT) </= 2.5 x ULN

7) Alkaline phosphatase </= 2.5 x ULN

8) Provide signed written informed consent.

9) Capable of understanding the investigational nature, potential risks and benefits of the study, and able to provide valid informed consent.

10) Female patients of childbearing potential must have a negative pregnancy test within 2 weeks prior to entering this study.

11) Women who are able to become pregnant and men who can father a child must use birth control while on study and for at least 8 weeks after your last dose of study drug(s). Acceptable birth control includes a condom or a diaphragm with spermicidal jelly; and birth control methods that are taken by mouth, injected, or implanted. If you are already using birth control, you must check with the study staff to make sure that it is considered one of the acceptable forms to use in this study.

Exclusion:

1) Current concomitant chemotherapy, radiation therapy, or immunotherapy other than as specified in the protocol.

2) Use of investigational agents within 30 days or any anticancer therapy within 2 weeks prior to entering this study with the exception of hydroxyurea. The patient must have recovered from all acute toxicities from any previous therapy.

3) Have any other severe concurrent disease, or have a history of serious organ dysfunction or disease involving the heart, kidney, liver, or other organ system that may place the patient at undue risk to undergo treatment.

4) Patients with a systemic fungal, bacterial, viral, or other infection not controlled (defined as exhibiting ongoing signs/symptoms related to the infection and without improvement, despite appropriate antibiotics or other treatment).

5) Pregnant or lactating patients.

6) Any significant concurrent disease, illness, or psychiatric disorder that would compromise patient safety or compliance, interfere with consent, study participation, follow up, or interpretation of study results.

7) Any concurrent malignancy (with the exception of exclusion # 8)

8) Exceptions to # 7: a) Patients with treated non-melanoma skin cancer, in situ carcinoma, or cervical intraepithelial neoplasia, regardless of the disease-free duration, are eligible for this study if definitive treatment for the condition has been completed; b) Patients with organ-confined prostate cancer with no evidence of recurrent or progressive disease based on prostate-specific antigen (PSA) values are also eligible for this study if hormonal therapy has been initiated or a radical prostatectomy has been performed.

4.0 Treatment Plan

4.1 Study Treatment

Patients will be treated with SGI-110 60mg/m^2 subcutaneously daily x 5 every 4 weeks until treatment failure or for a maximum of 2 years. Further therapy beyond 2 years could be extended if judged in the best patient interest. Treatment will be continued unless patients exhibit evidence of treatment failure, disease progression, experience an unacceptable toxicity, or the investigator determines that discontinuation of treatment is in the patient best interest.

All patients will be registered and entered through the Protocol Data Management System (PDMS).

4.2 Treatment Plan

- SGI-110 60mg/m^2 SQ daily x 5 days every 4 weeks for a total of 24 courses. Interval between courses can be extended to 8 weeks depending on toxicities, patient condition, and recovery of course.

- Patients should not be taken off therapy with SGI-110 prior to completion of the first 3 courses unless for unequivocal disease progression while on therapy, unacceptable toxicities, or patient request.

- Patients with stable disease after completion of three courses can continue for up to 6 courses. Unless patients have achieved at least a hematological improvement after 6 courses, they should then be taken off therapy with SGI-110.

- Patients may receive up to 24 courses depending on response and tolerability of therapy. Courses should be repeated every 4 to 8 weeks. Continuing of SGI-110 beyond 24 courses is an option if judged to be in the patient best interest.

4.2.1 Supportive Measures during Treatment

Necessary supportive measures for optimal medical care are to be given throughout the study as indicated by the treating physician's assessment of the patient's medical need and by the institutional guidelines as far as established.

4.3 Dose Modifications

4.3.1 Induction, Re-Induction, and Consolidation Cycles

All dose delays, reductions, and modifications for hematologic and non-hematologic toxicities will be assessed according to Section 9, and Table 5 and 6.

4.3.2 Hematologic (Blood/Bone Marrow) Toxicity

No dose reductions, delays, or modifications are required for hematologic toxicities during the first 2 cycles of treatment. It is assumed that low counts at diagnosis are due to involvement by the disease process and require therapy for improvement.

Patients who achieve a CR that have not recovered peripheral blood counts will be treated with subsequent cycles and other dose modifications implemented at the discretion of the investigator.

4.3.3 Non-Hematologic Toxicity

- Reduce SGI-110 by 25% for grade 3-4 reversible toxicities (same as above). Dose reduction for grade 2 reversible toxicities and other dose modifications can be implemented if in the best interest of the patient, after discussion with primary investigator.

4.4 Infection Prophylaxis

The use of prophylactic antibacterial (e.g., levaquin), antifungals (e.g., voriconazole), and antiviral agents (e.g., valacyclovir) is recommended according to institutional guidelines as established.

4.4.1 Colony Stimulating Factors

Hematopoietic growth factors (e.g., granulocyte colony stimulating factor [G-CSF], (erythropoietin) can be administered at the treating physician's discretion and judgment.

4.4.2 Concomitant Therapy

No concomitant cytotoxic therapy or investigational therapy is allowed during the study. Concomitant medications will be documented in the medical record.

5.0 Pretreatment Evaluations

History and physical, CBC with differential and platelets, chemistry profile (total bilirubin, serum creatinine, SGPT or SGOT, uric acid, LDH, potassium, magnesium) within 14 days of therapy start.

Bone marrow aspirate and/or biopsy within 28 days of therapy start. The bone marrow evaluation will include immunophenotyping by flow cytometry and cytogenetic studies.

Pregnancy (urine or blood) test for women of childbearing potential within two weeks prior of entering this study. Child bearing potential is defined as not post-menopausal for 12 months or no previous surgical sterilization.

Screening physical assessment does not need to be repeated on Cycle 1 Day 1, if performed within 2 days.

6.0 Evaluation during Study

- CBC with differential and platelet counts weekly in the first course, then every 2 to 8 weeks thereafter as long as on study. No differential is needed if the WBC is < 1.0 x 10^9/L.
- Chemistry profile (at least creatinine, SGOT or SGPT, total bilirubin) every 2-8 weeks while on therapy, then every 8 to 12 weeks as long as on study.
- Bone marrow aspirate and/or biopsy at the end of course 1 and/or 2 of SGI-110 (day 28 +/- 7 days). If a patient is not in remission at this point, further bone marrow aspirate and/or biopsy should be scheduled to document remission, for example at the end of course 6 (day 28 +/- 7 days).
- Bone marrow tests can be ordered more frequently if mandated by development of peripheral blood counts. No repeat bone marrow is necessary if non-response or progressive disease can be unequivocally diagnosed from peripheral blood tests or, in patients with a WBC $\leq$ 0.3 if the bone marrow test is considered non-contributory by the investigator at any time point.
- Follow up marrow tests should include cytogenetic analysis for those patients who had abnormalities prior to therapy start.
- Repeat physical examination (problem focused) on Day 1 ($\pm$ 3 days) of every cycle as long as on active therapy.
- After completion of therapy and/or 30 days after last dose of study drug, patients will be observed for survival only when off active treatment. Patients will be called 30 days after last dose of study drug and every 2 months (+/- 2 months) thereafter.
- Optional: Blood 10cc pretreatment and on Day 5 +/- 2 days and Day 14 +/- 2 days to evaluate effect of SGI-110.
- All outside labs will need to be reviewed, signed and dated by the Principal Investigator (PI) or treating physician (listed on the Delegation of Authority) prior to submission to the electronic medical record. The PI or treating physician should appraise abnormal lab results and determine/document clinical significance

7.0 Criteria for Response

1) Complete Remission:

Normalization of the peripheral blood and bone marrow with </= 5% bone marrow blasts, a peripheral blood granulocyte count >/= 1.0 x 10^9/L, and a platelet count >/= 100 x 10^9/L. Hgb >/= 11 g/dL at any point while on treatment after the first cycle of therapy.

Addition of CRi (Complete Remission without blood count recovery) = Peripheral blood and bone marrow counts as per CR, but with platelet counts 30-100 x 10^9/L and/or ANC < 1.0 x 10^9/L.

2) Partial Remission:

Patients must demonstrate all CR criteria if abnormal before treatment except that marrow blasts should decrease by 50% or more compared to pretreatment levels or may demonstrate a less-advanced MDS disease classification category than prior to treatment.

3) Hematologic Improvement:

Response maintained for at least 8 weeks:
- Hemoglobin (pretreatment < 11 g/dL): improves by 1.5 g/dL or reduced by 4 units of RBC transfusions in 8 week time period compared with pretreatment transfusion number in 8 weeks. Only RBC transfusion given for Hgb of </= 9.0 g/dL will count in the RBC transfusion response evaluation.
- Platelet (pretreatment < 100 x 10^9/L): absolute increase of >/= 30 x 10^9/L for patients starting with plt > 20 x 10^9/L OR increase from < 20 x 10^9/L to > 20 x 10^9/L and by at least 100%.
- Neutrophil (pretreatment < 1 x 10^9/L): increase 100% and absolute increase > 0.5 x 10^9/L

4) Clinical Benefit:

Platelets increase by 50% and to above 30 x 10^9/L untransfused (if lower than that pre-therapy); or granulocytes increase by 100% and to above 1 x 10^9/L (if lower than that pre-therapy); or hemoglobin increase by 2 g/dL; or transfusion independent; or splenomegaly reduction by >/= 50%; or monocytosis reduction by >/= 50% if pretreatment > 5 x 10^9/L.

8.0 Criteria for Removal from the Study

Reason to take patients off study include (but are not limited to) the following:

- Progressive disease
- Patient refusal
- Patient non-compliance
- Physician judgment

9.0 Statistical Considerations

This is an open-label, phase II study of SGI-110 in patients with higher risk MDS. The primary endpoint is complete response (CR) in patients with higher risk MDS treated with SGI-110. CR will be first evaluated at any time after the first cycle, then repeated every 3 cycles thereafter as long as the patient continues treatment. A maximum of 100 patients will be treated (3-5 per month).

The method of Thall, Simon, and Estey [19] will be used to futility, monitor toxicity (adverse events and mortality at 3 months). Multc Lean Desktop (version 2.0) was used to generate the stopping boundaries and the OC tables for futility and the command stopbound in Stata for the toxicity monitoring.

Futility Monitoring

The historical data suggested a complete response rate of 10% with the **standard of care treatment (AZA)** and the target response rate with the experimental treatment (SGI-110) is 25% . Given this, we will stop enrollment into this cohort if the observed patients' data suggest that:

$$Pr(\pi_E > \pi_H + 0.15 | data) < 0.025$$

Here π_E and π_H are the complete response rate (CRR) for the experimental treatment and historical standard of care treatment, respectively. That is, if at any time during the study we determine that there is a less than 2.5% chance that the average CRR improves over historical rate by more than 15% we will stop enrollment to this cohort. π_E and π_H are assumed to follow a prior of Beta (0.2, 1.8) and Beta(100, 900), respectively. The stopping boundaries for CRR, based on these assumptions and monitoring conditions are found in **Table 3.** We will apply these stopping boundaries in cohorts of 10 starting from the 10th patient. For example, accrual will cease if 0 patients experiences complete response among the first 10 patients treated. The operating characteristics are summarized in **Table 4.**

Table 3. Stopping boundaries for CRR

Number of patients evaluated for complete response	10	20	30	40	50	60	70	80	90
Number of patients with complete response is less than or equal to	0	0-1	0-3	0-5	0-7	0-9	0-11	0-13	0-15

Table 4. Operating characteristics for monitoring CRR

True Complete Response Rate	Early Stopping Probability	Average number of patients treated
0.10	0.9908	28
0.15	0.8238	50
0.20	0.4285	75
0.25	0.1476	90
0.30	0.0464	96
0.35	0.0166	99

Toxicity Monitoring

In addition, we will monitor toxicities (i.e., Grade≥3 non-hematological toxicity) for all the patients treated. The toxicities will be evaluated **at the end of week 12 from the start of treatment.** The probability of toxicity is denoted by P_E. We assume P_E ~ beta (0.6, 1.4). Our stopping rule is given by the following probability statement: $Pr(P_E > 0.30 | data) > 0.95$. That is, we will stop the study if, at any time during the

study, we determine that there is more than 95% chance that the toxicity is more than 30%. The stopping boundaries for toxicities, based on these assumptions and monitoring conditions is found in **Table 5.** We will apply the toxicity monitoring rule in cohort size of 5 (minimum one patient). For example, accrual will cease if 4 patients experience toxicities among the first 5 patients treated. The operating characteristics are summarized in **Table 6.**

Table 5. Stopping boundaries for toxicity monitoring

The number of patients evaluated for toxicities	5	10	15	20	25	30	35	40	45	50	55	60	65	70	75	80	85	90	95
The number of patients with toxicities is greater than or equal to	4	6	8	10	12	14	16	18	19	21	23	25	26	28	30	32	33	35	37

Table 6. Operating characteristics for toxicity monitoring

True Toxicity Rate	Early Stopping Probability	Sample Size		
		25th percentile	Median	75th percentile
0.10	0.0008	100	100	100
0.20	0.0158	100	100	100
0.30	0.1879	100	100	100
0.40	0.7741	15	45	90
0.50	0.9947	10	15	30

Mortality rate at 3 months monitoring

In addition, we will monitor the mortality rate at 3 months for all the patients treated. The probability of mortality is denoted by P_E. We assume P_E ~ beta (0.2, 1.8). Our stopping rule is given by the following probability statement: $Pr(P_E > 0.10 \mid data)>0.95$. That is, we will stop the study if, at any time during the study, we determine that there is more than 95% chance that the mortality rate is more than 10%. The stopping boundaries for mortality rate at 3 months, based on these assumptions and monitoring conditions is found in **Table 7.** We will apply the mortality monitoring rule in cohort size of 10 (minimum one patient). For example, accrual will cease if all 3 patients died among the first 5 patients treated. The operating characteristics are summarized in **Table 8.**

Table 7. Stopping boundaries for mortality monitoring

The number of patients evaluated for mortality	5	10	15	20	25	30	35	40	45	50	55	60	65	70	75	80	85	90	95
The number of patients dead is greater than or equal to	3	4	5	5	6	7	8	8	9	10	10	11	12	12	13	14	14	15	15

Table 8. Operating characteristics for mortality monitoring

True Mortality Rate	Early Stopping Probability	Sample Size		
		25th percentile	Median	75th percentile
0.06	0.0111	100	100	100
0.08	0.0444	100	100	100
0.10	0.1291	100	100	100
0.12	0.2751	85	100	100
0.14	0.4759	40	100	100

Statistical Analysis Plan

All patients who received at least 1 dose of the experimental treatment will be included in the intent-to-treat analysis for efficacy and safety. Safety data will be summarized using frequency and percentage, by organ type, grade and attribution. Complete response and overall response rates will be estimated along with the 95% credible intervals. Survival times (overall survival, time to AML transformation and event-free survival) will be estimated using the Kaplan-Meier method for each patient cohort.

10.0 Reporting Requirements

Serious adverse events (SAEs) considered associated with therapy should be reported to the Principal Investigator [Guillermo Garcia-Manero, M.D., Telephone (713) 745-3428] within 24 hours of observing or learning of the event. All AEs should be reported to the research nurse. The principal investigator, in turn, will be responsible for reporting the event to the IRB, FDA, and the SGI-110 manufacturer (Astex Pharmaceuticals, Inc.) as applicable in compliance with regulatory and institutional requirements. Adverse events will be graded using CTCAE version 4.03 (Appendix D)

Serious and unexpected adverse events will be reported accordingly to MDACC guidelines. (Appendix C entitled "Leukemia Specific AE Recording Guidelines (09/2009)-Leukemia specific Adverse Event Recording and Reporting Guidelines". These events will be reported to the study chairman, who in turn will notify the Clinical Research Compliance office and the MDACC IRB. The Clinical Research Compliance Office will be responsible to notify the FDA according to 21CFR312.32.

- The investigator or physician designee is responsible for verifying and providing source documentation for all adverse events and assigning the attribution for each event for all subjects enrolled on the trial.
- Grade 3 or 4 related and/or unexpected adverse events and protocol specific data will be entered into PDMS/CORe. PDMS/CORe will be used as the electronic case report form for this protocol.

SERIOUS ADVERSE EVENT REPORTING FOR MD ANDERSON – IND SPONSORED PROTOCOLS

Serious Adverse Event Reporting (SAE)

An adverse event or suspected adverse reaction is considered “serious” if, in the view of either the investigator or the sponsor, it results in any of the following outcomes:

- Death
- A life-threatening adverse drug experience – any adverse experience that places the patient, in the view of the initial reporter, at immediate risk of death from the adverse experience as it occurred. It does not include an adverse experience that, had it occurred in a more severe form, might have caused death.
- Inpatient hospitalization or prolongation of existing hospitalization
- A persistent or significant incapacity or substantial disruption of the ability to conduct normal life functions.
- A congenital anomaly/birth defect.

Important medical events that may not result in death, be life-threatening, or require hospitalization may be considered a serious adverse drug experience when, based upon appropriate medical judgment, they may jeopardize the patient or subject and may require medical or surgical intervention to prevent one of the outcomes listed in this definition. Examples of such medical events include allergic bronchospasm requiring intensive treatment in an emergency room or at home, blood dyscrasias or convulsions that do not result in inpatient hospitalization, or the development of drug dependency or drug abuse (21 CFR 312.32).

- **Important medical events as defined above, may also be considered serious adverse events. Any important medical event can and should be reported as an SAE if deemed appropriate by the Principal Investigator or the IND Sponsor, IND Office.**

- All events occurring during the conduct of a protocol and meeting the definition of a SAE must be reported to the IRB in accordance with the timeframes and procedures outlined in “The University of Texas M. D. Anderson Cancer Center Institutional Review Board Policy for Investigators on Reporting Unanticipated Adverse Events for Drugs and Devices”. Unless stated otherwise in the protocol, all SAEs, expected or unexpected, must be reported to the IND Office, regardless of attribution (within 5 working days of knowledge of the event).

- **All life-threatening or fatal events,** that are unexpected, and related to the study drug, must have a written report submitted within **24 hours** (next working day) of knowledge of the event to the Safety Project Manager in the IND Office.

- **Unless otherwise noted, the electronic SAE application (eSAE) will be utilized for safety reporting to the IND Office and MDACC IRB.**
- **Serious adverse events will be captured from the time of the first protocol-specific intervention, until 30 days after the last dose of drug, unless the participant withdraws consent. Serious adverse events must be followed until clinical recovery is complete and laboratory tests have returned to baseline, progression of the event has stabilized, or there has been acceptable resolution of the event.**

- **Additionally, any serious adverse events that occur after the 30 day time period that are related to the study treatment must be reported to the IND Office. This may include the development of a secondary malignancy.**

Reporting to FDA

Serious adverse events will be forwarded to FDA by the IND Sponsor (Safety Project Manager IND Office) according to 21 CFR 312.32.

It is the responsibility of the PI and the research team to ensure serious adverse events are reported according to the Code of Federal Regulations, Good Clinical Practices, the protocol guidelines, the sponsor's guidelines, and Institutional Review Board policy.

Investigator Communication with Supporting Companies:

Reporting to Astex Drug Safety.

The Investigator will notify Astex, as designated holder of the global safety database, of any Serious Adverse Event (SAE), pregnancy, or infant exposure, including those events reported to the Investigator by his/her Investigators, whether related to Study Drug or not, within twenty-four (24) hours of being made aware of the SAE. Such notification will be provided to Astex by faxing the information contained in the exemplar Serious Adverse Event Report Form, which is appended to the Protocol, to the Astex Drug Safety Department:

Astex Drug Safety SAE Reporting Fax Number

Local Fax: 925-551-3226
North America Toll-Free Fax: 800-576-6568

A copy of the correspondence sent to the FDA [or other controlling regulatory authority] shall be sent within twenty-four (24) hours to Astex Regulatory Affairs by email or fax:

Astex Regulatory Affairs email: david.smith@astx.com

Astex Regulatory Affairs Fax Number: 925.551.6491

Disputed SAEs. Even if the Investigator decides not to report an event, Astex may make its own determination that it is a reportable Serious Adverse Event, and may report it.

11. References

1) Cheson BD, et al. Report of an international working group to standardize response criteria in myelodysplastic syndromes. Blood 2000; 96: 3671-3674.
2) Heany ML, Golde DW. Myelodysplasia. N Engl J Med 1999; 340: 1649-1660.
3) Bennett JM, et al. Proposals for the classification of the myelodysplastic syndromes. Br J Haematol 1982; 51: 2499-2509.
4) Kantarijan HM, Estey E. Myelodysplastic syndromes. Lippincott Williams & Wilkins, Eds. DeVita VT, Hellman S, Rosenberg SA, CANCER, 6th Edition, pp 2499-2509, 2001.
5) Greenberg P, et al. International scoring system for evaluating prognosis in myelodysplastic syndromes. Blood 1997; 89: 2079-2088.
6) Silverman LR et al. Randomized controlled trial of azacitidine in patients with myelodysplastic syndrome: a study of the Cancer and Leukemia Group B. J Clin Oncol 2002; 20: 2429-2440.
7) Fenaux P, et al. Efficacy of azacitidine compared with that of conventional care regimens in the treatment of higher-risk myelodysplastic syndromes: a randomized, open-label, phase III study. Lancet Oncology 2009; 19: 223-232
8) Santini V, et al. Changes in DNA methylation in neoplasia: pathophysiology and therapeutic implications. Ann Intern Med 2001; 134: 573-586.
9) Wijermans P, Lubbert M, Verhoef G, et al. Low-dose 5-aza-2'-deoxycytidine, a DNA hypomethylating agent, for treatment of high-risk myelodysplastic syndrome: a multicenter phase II study in elderly patients. J Clin Oncol 2000; 18: 956-962.
10) Kantarjian H, et al. Decitabine improves patient outcomes in myelodysplastic syndrome. Cancer 2006; 106: 1794-1803.
11) Wijermans P., et.al. Low dose decitabine versus best supportive care in elderly patients with intermediate or high risk MDS not eligible for intensive chemotherapy: Final results of the randomized Phase III Study (06011) of the EORTC Leukemia and German MDS Study Groups. ASH Annual Meeting Abstracts, 2008. 112(11): Abstract 226.
12) Kantarjian H., et al. Results of a randomized study of 3 schedules of low-dose decitabine in higher-risk myelodysplastic syndrome and chronic myelomonocytic leukemia. Blood, 2007: 109(1): 52-57.
13) Steensma DP, et.al. Multicenter study of decitabine administered daily for 5 days every 4 weeks to adults with myelodysplastic syndromes: the alternative dosing for outpatient treatment (ADOPT) trial. J Clin Oncol, 2009: 27(23): 3842-3848.
14) Kantarjian, H., et al. Results from the dose escalation phase of a randomized Phase 1-2 first-in-human (FIH) study of SGI-110, a novel low volume stable subcutaneous (SQ) second generation hypomethylating agent (HMA) in patients with relapsed/refractory MDS and AML. ASH Annual Meeting Abstracts, 2012: Abstract 414.
15) Kantarjian H, et al. First clinical results of a randomized phase 2 study of SGI-110, a novel subcutaneous (SQ) hypomethylating agent (HMA), in adult patients with acute myeloid leukemia (AML). Submitted to ASH 2013 (Confidential-has been submitted to ASH 2013)
16) Thall PF, Simon RM, Estey EH. Bayesian sequential monitoring designs for single-arm clinical trials with multiple outcomes. Stat Med. 1995 Feb 28;14(4):357-79.
