# Supplementary material for: Prospective performance of the IWG-2023 criteria and IPSS-M in a phase 2 trial of guadecitabine for higher-risk MDS or CMML
Source: Blood Neoplasia. 2024 Mar 29;1(2):100008. doi: 10.1016/j.bneo.2024.100008 (PMC12082099; doi:10.1016/j.bneo.2024.100008)
Supplement: Supplemental Figures [file BNEO_NEO-2024-000211-mmc3.pdf]

Supplemental Figures

Figure S1 - Mutational profile of patients with MDS..2
Figure S2 - Mutational profile of patients with CMML...3
Figure S3 - Mutational profile by CMML IWG 2023 criteria 4
Figure S4 - OS duration by IPSS-M 5
Figure S5 – IPSS-R mapping to IPSS-M...6
Figure S6 – IPSS classifications at diagnosis...7
Figure S7. Forrest plot for ORR by IWG 2023...8
Figure S8. OS by IWG 2006 and IWG 2023. 9

Figure S1 - Mutational profile of patients with MDS

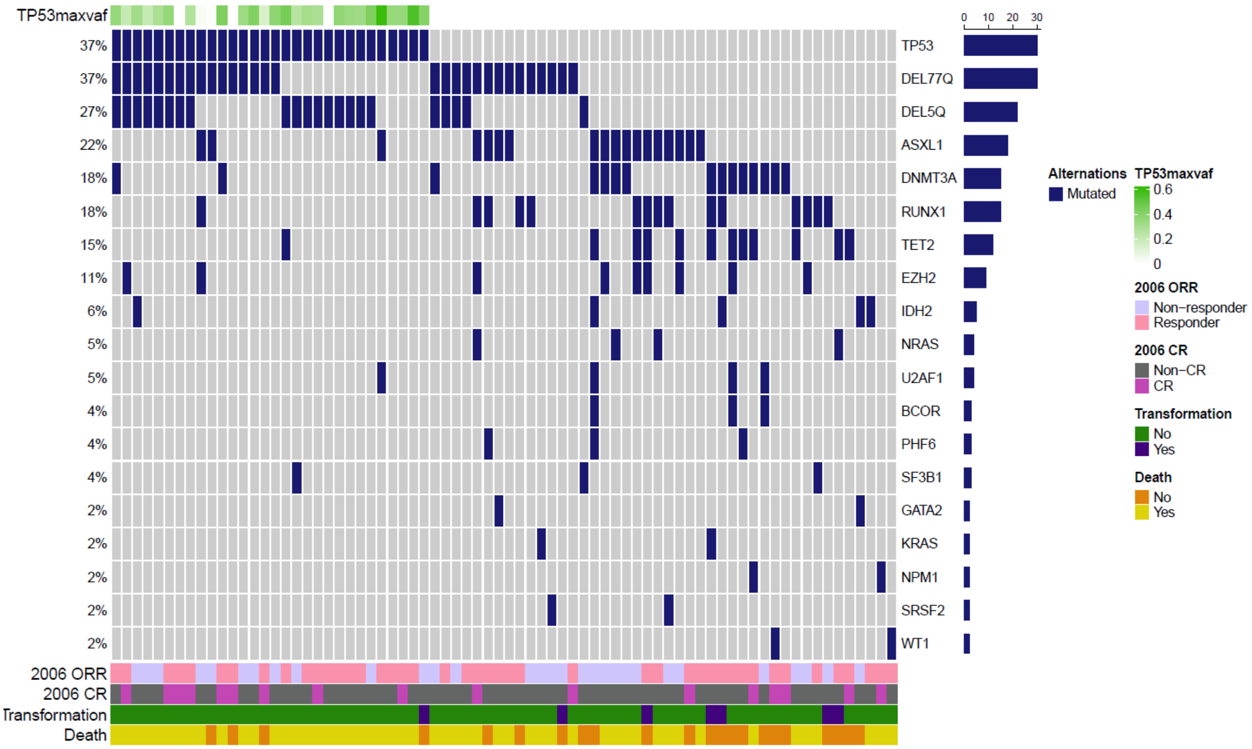

Figure S1. Mutational profile of MDS patients including *TP53* VAF and response by IWG-2006 criteria, AML transformation or death.

Figure S2 - Mutational profile of patients with CMML

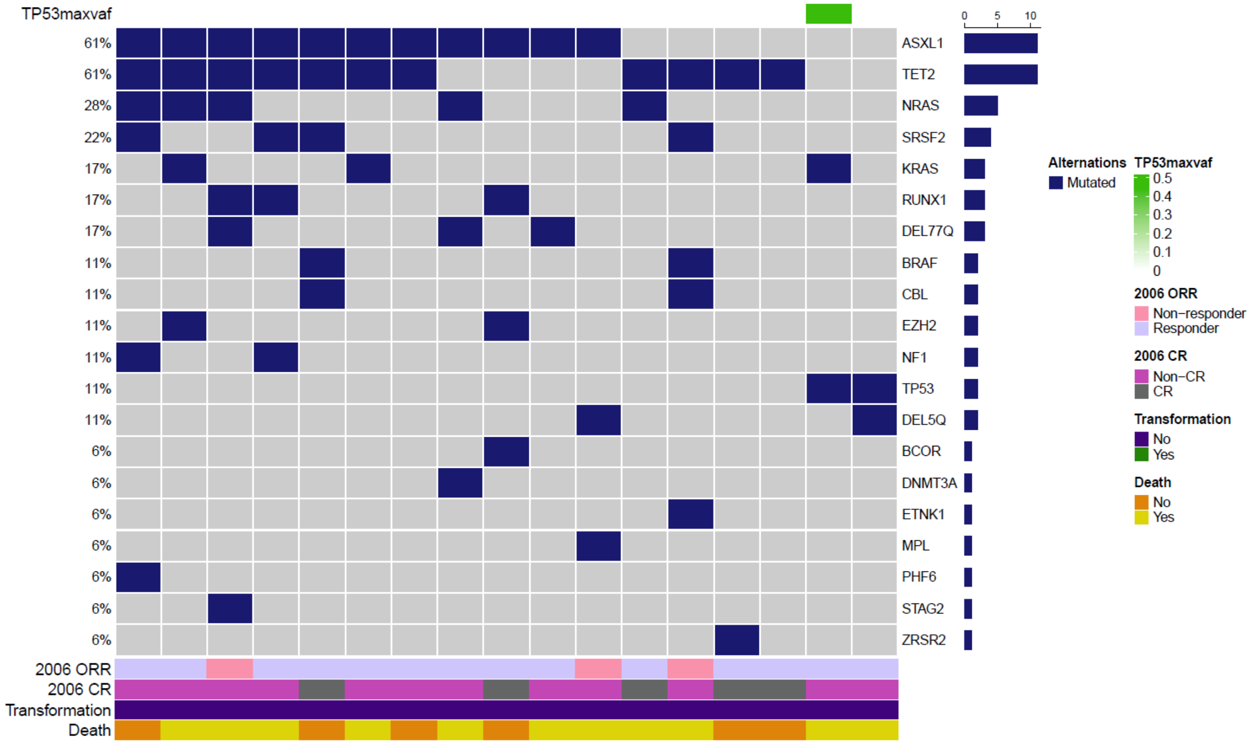

Figure S2. Mutational profile of CMML patients response by IWG-2006 criteria, AML transformation or death.

Figure S3 - Mutational profile by CMML IWG 2023 criteria

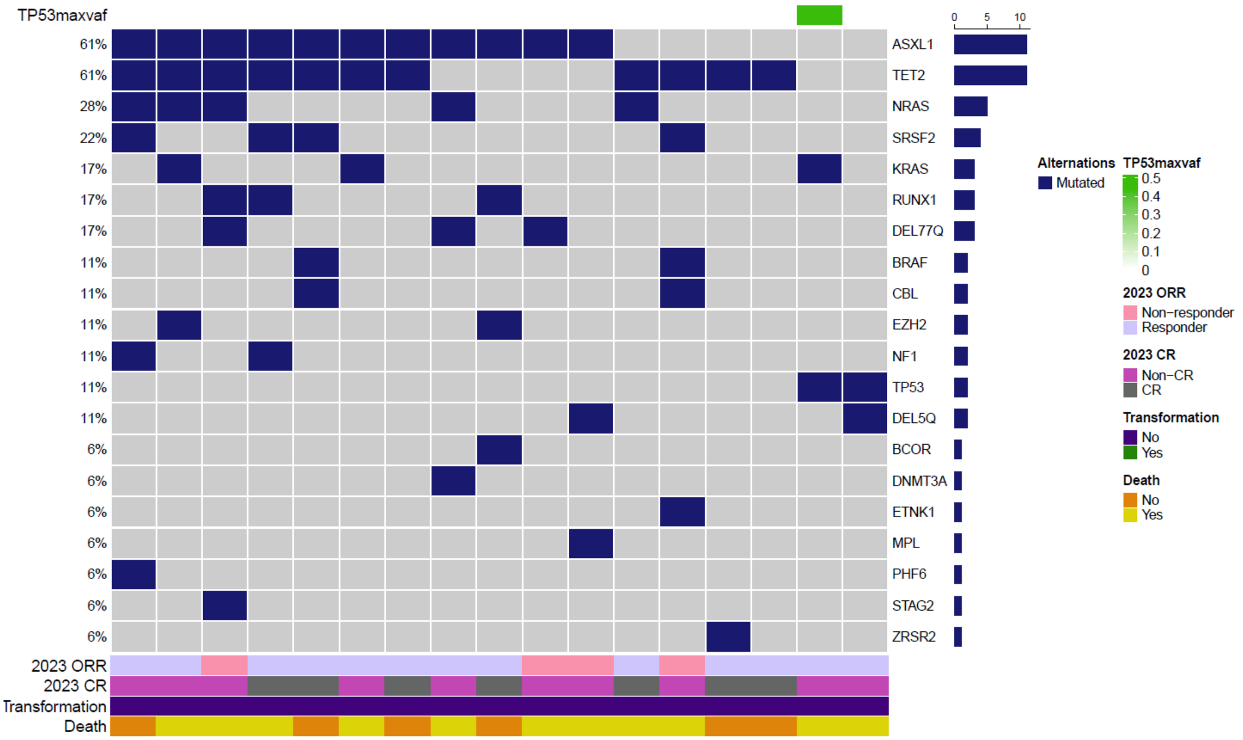

Figure S3. Mutational profile of CMML patients response by IWG-2023 criteria, AML transformation or death.

Figure S4 - OS duration by IPSS-M

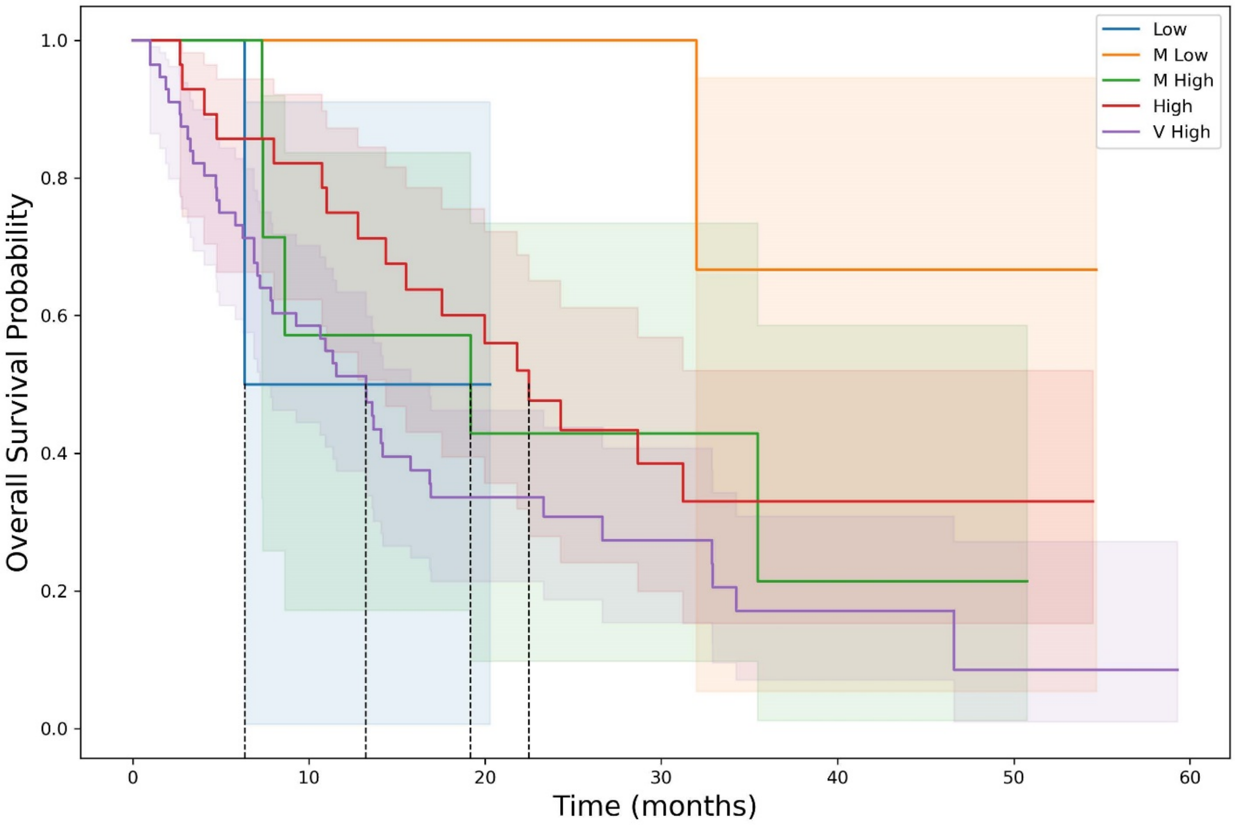

At risk							
Low	2	1	1	0	0	0	0
M Low	7	7	5	5	2	1	0
M High	7	4	3	2	1	1	0
High	28	23	14	7	2	1	0
V High	56	32	15	8	3	1	0

Figure S4. Overall survival by IPSS-M classification at diagnosis.

Figure S5 – IPSS-R mapping to IPSS-M

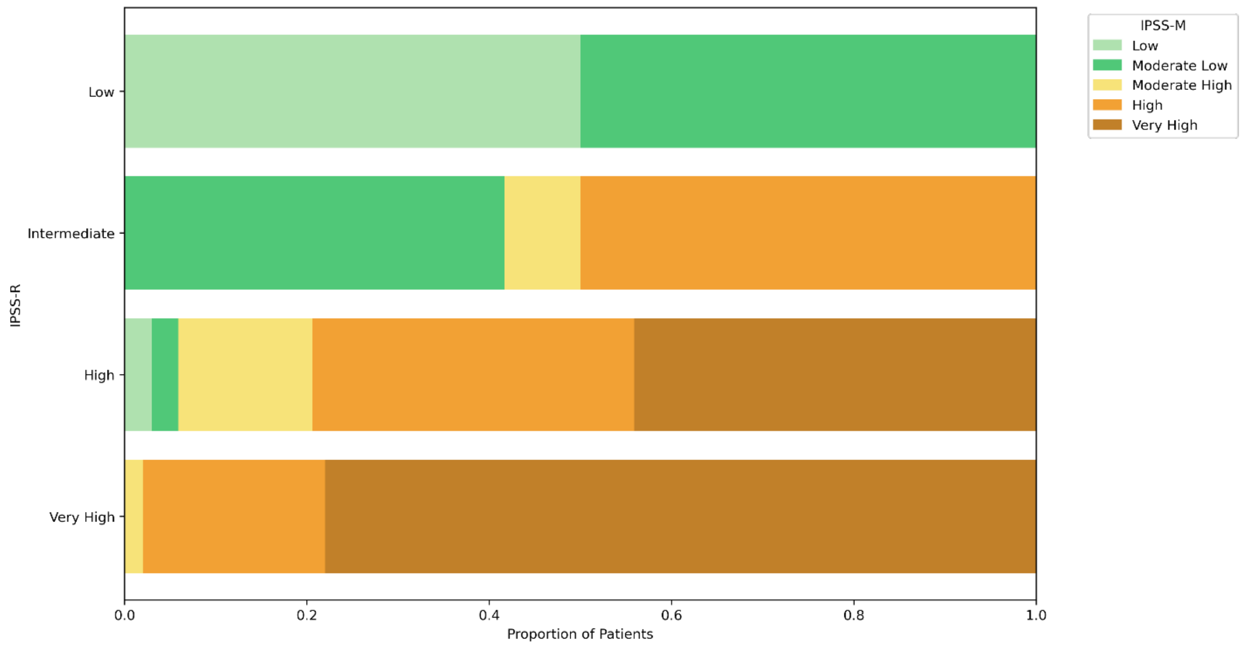

Figure S5. IPSS-R categories in the Y axis vs proportion of patients with IPSS-M scores in the X axis.

Figure S6 – IPSS classifications at diagnosis

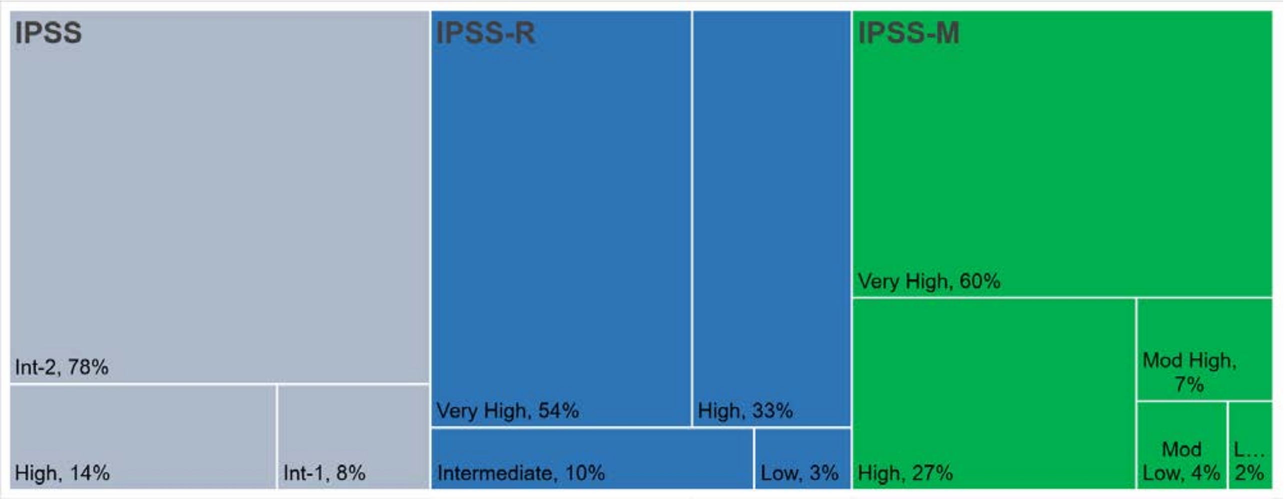

Figure S8. Treemap of classification distribution by IPSS, IPSS-R and IPSS-M.

Figure S7. Forrest plot for ORR by IWG 2023.

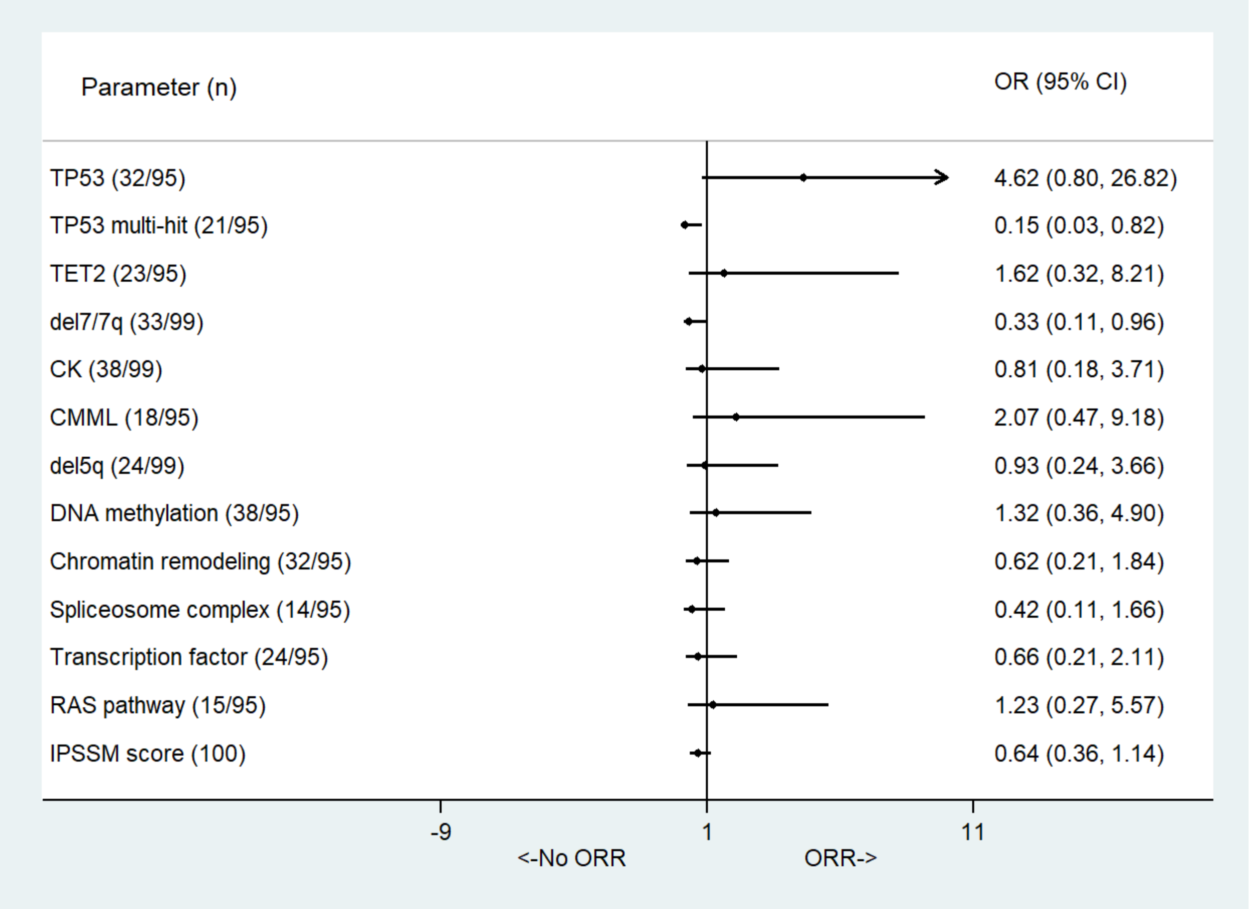

Figure S7. Forest plot of odds ratio of overall response rate by International Working Group 2023 criteria. CK: complex karyotype. CMML: chronic myelomonocytic leukemia. Pathways as grouped by Ogawa, et. al.[27]

Figure S8. OS by IWG 2006 and IWG 2023.

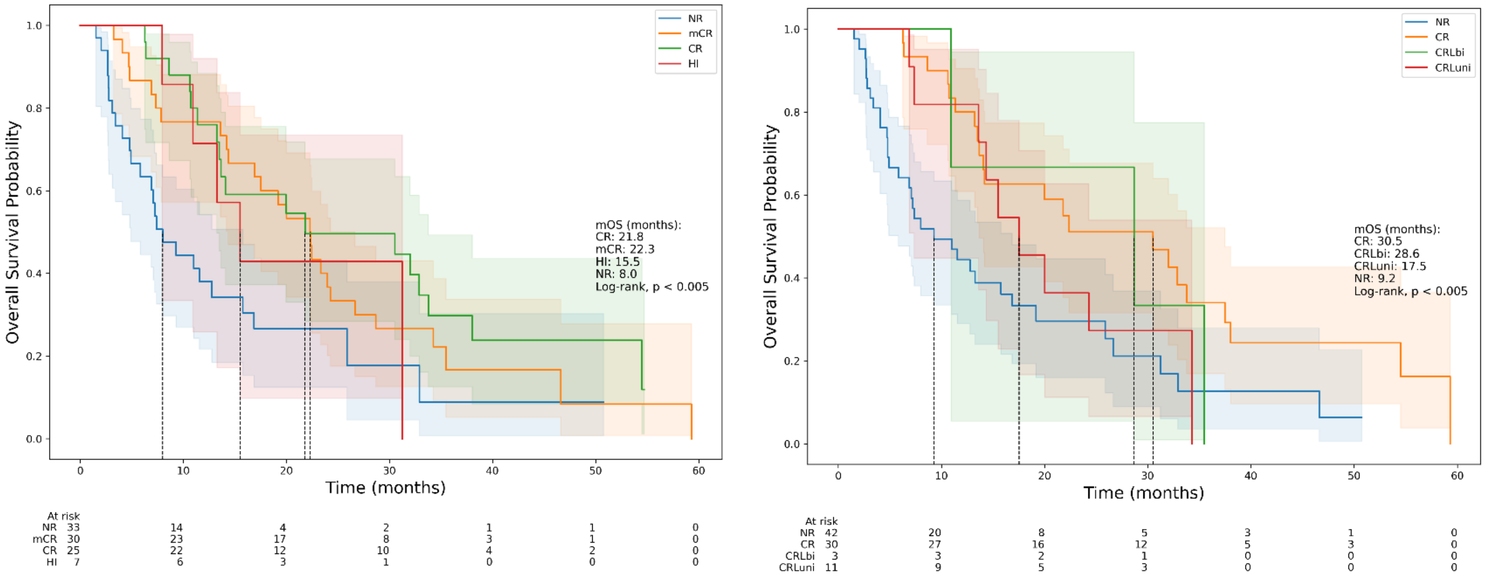

Figure S8. Kaplan Meier overall survival probabilities by IWG 2006 (left) and IWG 2023 criteria. CR: complete response, CRLbi: limited CR with bilineage recovery, CRLuni: limited CR with unilineage recovery, mCR: marrow complete response, NR: no response. Other categories not included due to n < 5.
